# Spatial Analysis and Lead‐Risk Assessment of Philadelphia, USA

**DOI:** 10.1029/2021GH000519

**Published:** 2022-03-01

**Authors:** H. Caballero‐Gómez, H. K. White, M. J. O’Shea, R. Pepino, M. Howarth, R. Gieré

**Affiliations:** ^1^ Department of Chemistry Haverford College Haverford PA USA; ^2^ Now at University of California Los Angeles CA USA; ^3^ Department of Earth and Environmental Science University of Pennsylvania Philadelphia PA USA; ^4^ Center of Excellence in Environmental Toxicology University of Pennsylvania Philadelphia PA USA

## Abstract

Childhood lead poisoning is an issue that continues to plague major U.S. cities. Despite efforts by the Philadelphia Department of Public Health to curtail systemic childhood lead poisoning, children continue to be identified with elevated blood lead levels. The persistence of elevated blood lead levels in children is concerning because lead poisoning has been linked to decreases in academic achievement and IQ, with associated repercussions for entire communities. This paper reports the results of an analysis of the spatial distribution of houses with lead paint (i.e., pre‐1978), demolitions, and occurrence of historic smelters, in West and North Philadelphia, relative to elevated blood lead level data, to determine which lead sources act as primary lead‐risk factors. The presence of lead paint in homes and the number of demolitions of older properties were found to have the highest correlations to elevated blood lead levels for children in Philadelphia. Using lead‐risk factors including lead paint, housing code violations, demolitions, and owner‐occupied housing units, a lead‐risk assessment was performed at the census tract level to identify future soil sampling sites and high‐risk neighborhoods in Philadelphia. These sites of high risk for lead exposure, and in particular the census tracts 175 and 172, should be prioritized for lead poisoning prevention initiatives.

## Key Points

- In areas with high homeownership, owner‐occupied units can serve as lead‐risk indicators for childhood lead poisoning
- Demolitions and lead paint of older housing are critical lead‐source emissions for childhood lead poisoning in Philadelphia
- North Philadelphia soils, and especially open‐land around historic smelters, are highly undersampled

## 1 Introduction

The toxicity of lead and its extensive use in human activities have made lead pollution a pressing health issue worldwide (Agency for Toxic Substances and Disease Registry ATSDR, 2020). The presence of lead is especially concerning in industrial and post‐industrial areas, due to the heavy metal's damaging effects on human health. Lead can be absorbed into the body via inhalation, ingestion, and to a limited extent dermally. However, lead has no biological function in the body and, once absorbed, it has a multitude of detrimental effects on health, including anemia, hypertension, renal and cognitive impairments (Aoki et al., 2017; Hauptman et al., 2017; International Agency for Research on Cancer; IARC, 2006; Sergio & Sordo, 2006; Wani et al., 2015; World Health Organization; WHO, 2021). Significantly, there is no safe level of exposure to lead (Bellinger, 2008; WHO, 2021). The toxicity of lead is more pronounced in children 0–6 years of age so that, even at a relatively low concentration of 5 μg/dL of lead in blood, children begin to experience decreases in IQ and academic achievement (Aoki et al., 2017; Bellinger, 2008; Hauptman et al., 2017; Wani et al., 2015). Therefore, children are most vulnerable to lead poisoning, and they are also at greater risk of exposure than adults due to their high rate of hand‐to‐mouth activity (Hauptman et al., 2017).

Since the Consumer Product Safety Act of 1977, later amended by the Consumer Product Safety Improvement Act of 2008, United States (U.S.) legislation has decreased lead concentration in paints and similar coatings so that it must not exceed 90 ppm (WHO, 2020). In general, however, 1978 is understood as the year in which lead‐based paint was banned for residential use (Centers for Disease Control and Prevention CDC, 2020). Similarly, there have been active efforts at the local levels to curtail childhood lead poisoning (Aoki et al., 2017; IARC, 2006). In recent years, the Philadelphia Department of Public Health has reported an increase in the number of children screened alongside a decrease in the number of screened children with elevated blood lead levels (EBLLs). Despite this progress, however, significant numbers of children continue to be burdened by lead exposures. In 2018, the City of Philadelphia reported 1,434 newly identified children with blood lead levels (BLLs) at 5–9 μg/dL and 433 children with BLLs greater than 10 μg/dL (Philadelphia Department of Public Health, 2018). However, due to the CDC recently redefining the blood lead reference value from 5 to 3.5 μg/dL or greater (CDC, 2021a, 2021b), we expect the number of identified children with EBLLs to increase.

The majority of the City of Philadelphia is considered at risk for lead hazards from lead paint but the issue of childhood lead poisoning is highly racist and classist (Sicotte & Swanson, 2007). In five zip codes within low‐income communities of color in North and West Philadelphia, one in 15 children screened has EBLLs (PCCY, 2018). Another study found that children in parts of North Philadelphia, where the population is predominantly Black, were 2–2.5 times more likely to be lead poisoned than in the City of Philadelphia as a whole (Rothman et al., 2002). These studies highlight the disproportionate burden from pollutants seen in environmental justice communities, defined by the Pennsylvania Department of Environmental Protection (PA‐DEP) as having ≥20% of the population below the poverty level and/or ≥ 30% minority population, as is the case in parts of North Philadelphia (PA‐DEP, 2021a; Whitehead & Buchanan, 2019). At the national level, Black children experience 2.8 times higher odds of having BLLs >5 μg/dL even when compared to low‐income white and Latine populations, along with a greater likelihood of being exposed to lead (Yeter et al., 2020). In fact, according to Yeter et al. (2020), being Black is the second strongest predictor for EBLLs aside from housing built in 1950. A combination of historically racist redlining, poor housing quality, environmental racism, current inequitable enforcement of regulations, and poor healthcare access have compounded to create the current disparity in childhood lead poisoning (Sicotte & Swanson, 2007; Yeter et al., 2020).

Considering historic and current lead and lead‐contaminated dust production in Philadelphia, there are five major sources or processes that can continue to generate and disperse lead particles or that persist as lead residues in the environment. The five major lead sources (Figure S1 in the Supporting Information S1) are historic smelters, leaded gasoline, lead paint, demolitions, and lead pipes. Leaded gasoline was widely used in the U.S. starting in the 1920s until, as for paint, legislation mandated a significant decrease in lead concentration in 1978; however, during its use, it created large amounts of ambient lead (IARC, 2006), which is persistent and can still contribute significantly to the lead burden in cities (Resongles et al., 2021). Lead paint was both produced and used extensively in homes and on roads within Philadelphia from the late 1800s until 1978 (Aoki et al., 2017; Mielke & Reagan, 1998). Previous work has found high correlations between housing code violations, old property, and lead hazards within the home due to chipping lead paint and lead‐contaminated dust (Aoki et al., 2017; HUD, 2002; Lusby et al., 2015; Sergio & Sordo, 2006). Research completed in Philadelphia identified floor dust at household entryways as an important indicator of lead exposure in children (Dignam et al., 2019). Studies have also found an increase in lead‐contaminated dust and lead‐in‐soil near demolition and smelter sites (Farfel et al., 2003; Lauer, 2019; Lusby et al., 2015; Rabito et al., 2007; Rieuwerts & Farago, 1995; Sullivan, 2015; West Chester University, 2015). There is also evidence of significant lead‐contaminated dust accumulation on ceilings and roofs that, when disturbed during demolition, could resuspend hazardous lead‐contaminated particles into the air, nearby homes, streets, and soils (Davis & Gulson, 2005). Regarding lead drinking water pipes, also known as lead service lines, most cities in the U.S. stopped the production of such pipes in 1920, but Philadelphia was one of the few cities that continued installing and preserving lead pipes until 1950 (Rabin, 2008). For this reason, it is believed that housing units built before 1950 are more likely to have lead pipes, and therefore produce lead hazards.

With the exception of the lead pipes, the major sources of lead generate and disperse lead‐containing particles in air from where they settle and accumulate in soil or on other surfaces (e.g., roads, floors), either directly or subsequent to chemical and physical alteration. Because lead strongly adsorbs onto soil, it is a persistent environmental pollutant, which creates an ongoing risk of exposure, particularly in soils that have not been remediated (Hauptman et al., 2017; Sullivan, 2015; West Chester University, 2015). Soil is therefore the primary outdoor environmental medium to sample and analyze to identify lead presence and risk (Mielke & Reagan, 1998).

The approach in this study was developed with the goal of addressing existing health disparities in childhood lead poisoning. In order to do so, this work begins by investigating which lead sources continue to be lead‐risk factors in Philadelphia. Second, we verify which lead‐risk factors have the greatest impact on childhood lead poisoning in Philadelphia. Third, we determine which census tracts are at the highest risk for childhood lead poisoning, and identify sites for future soil sampling and analysis. In addition to lead sources and demographic data, evidence of lead such as lead‐in‐soil data, EBLLs, and brownfield/land recycled sites are considered for lead‐risk analysis. This study improves understanding of lead sources and spatial risk of poisoning in Philadelphia so that public health policy can provide comprehensive solutions aimed at preventing childhood lead poisoning, especially for at‐risk populations.

## 2 Materials and Methods

### 2.1 Study Area

The study area, broadly defined as West, North, and Upper North Philadelphia, was based on the results of a previous investigation, which analyzed lead risk at the zip code level within Philadelphia (O’Shea et al., 2021). Lead risk at the zip code level was determined by mapping various lead‐risk factors, including owner‐occupied units, renter‐occupied units, units built before 1980, demolitions, minority population (defined as racial and ethnic minorities within the United States), Black population, children in poverty, median income, EBLLs in children, number of smelters, and lead‐in‐soil data (O’Shea et al., 2021). A housing unit, as defined by the Census Bureau, is a house, an apartment, a group of rooms, or a single room intended to serve as a living quarter. Demolitions, as defined by OpenDataPhilly (2021), are all types of demolitions that are legally occurring throughout the city. The zip codes with the highest risk, that is, a high number of simultaneous lead‐risk factors, as identified by O’Shea et al. (2021) were zip codes 19121, 19125, 19132, 19133, 19134, 19138, 19140, 19141, 19143, and 19144 (Figure S2 in the Supporting Information S1; see also O’Shea et al., 2021). Zip codes 19134 and 19125, however, were omitted from being a part of the area investigated here due to significant remediation work and research carried out in this region.

For the study presented here, we evaluated ninety‐four census tracts (Figure S3 in the Supporting Information S1), all of which are located within each of the high‐lead‐risk zip codes (Table S1 and Figure S3 in the Supporting Information S1). Census tracts 9800, 9801, 9805, and 9809, also located in the high‐lead‐risk zip codes, were removed from the analysis as no data were found for these census tracts for various lead‐risk factors analyzed. Census tracts 71 and 172 are represented as a single census tract instead of two separate census tracts (e.g., 71.01 and 71.02 designated as 71) in the following analysis and maps, because data were represented in both forms across the sources of data, likely due to data being from different years and therefore census tract borders changing.

### 2.2 Lead‐Risk Assessment

Thirteen lead‐risk factors (Table 1) were evaluated to determine lead‐exposure risk within the region of study. The considered lead‐risk factors fall into three categories: (a) evidence of lead or lead exposure; (b) potential sources of lead; and (c) demographic risk factors as established by the CDC (CDC, 2019). Although lead exposure from all sources is cumulative, exposure sources vary for individuals. Our study is not designed to explore the relative contribution of lead exposure to individuals. Our grouping of risk factors is not intended to imply parity in their relative contribution, but rather represents a quantification of cumulative sources that combine to increase the risk of lead exposure.

**Table 1:** Lead‐Risk Categories and the Corresponding Lead‐Risk Factors Explored in This Study

Lead‐risk categories	Lead‐risk factors
Evidence of Lead or Lead Exposure	Elevated lead‐in‐Soil Data, EBLLs in Children, Brownfield/Land Recycled sites
Potential Lead Sources	Housing Code Violations, Critical Housing Code Violations, Lead Violations, Demolitions, Demolitions due to a Housing Code Violation, Properties Built Before 1980, Properties Built Before 1950, Smelters and manufacturing sites
Demographics previously associated with Lead‐risk Factors	Low Income, Minority Population

Despite the CDC's recently lowered blood lead reference of 3.5 μg/dL, this research reports and analyzes lead risk using the previous reference level of 5 μg/dL due to the availability of data at the time. Due to a lack of information regarding the location of lead pipes in Philadelphia, housing built before 1950 is used here as an indicator of the presence of lead pipes within a home. Because of conflicting results, renter‐occupied units were not included as a lead‐risk factor in our assessment of the lead risk, as described in Section 4.1 of this paper. However, the renter‐ and owner‐occupied housing data were analyzed since renter‐occupied housing units are typically regarded as a lead‐risk factor (CDC, 2019). Although leaded gasoline was identified as a lead source in Philadelphia considering the thousands of metric tons of lead estimated to have been deposited in Philadelphia soils, no data were found to analyze at the census tract level, and therefore our research did not include this lead source in its analysis. Critical housing code violations are factors that either accelerate paint deterioration or are linked to or produce lead‐paint hazards. Housing code violations labeled as critical housing code violations in this research are: lack of rental property license, lacking or poor debris removal, hazards due to poor plumbing system, poor ventilation, partial collapse of roof/wall, deterioration of roof/wall, or presence of cracked walls, and hazards due to unsafe interior or presence of lead. A lack of rental property license is linked to higher likelihood of lead hazards within the home (HUD, 2002). Meanwhile, poor ventilation and leaks from the plumbing system accelerate the deterioration of paint, whereas deterioration, cracking, and collapsing of walls produce lead paint chips and debris. For each census tract, the median lead‐in‐soil concentration was calculated. Then census tracts at highest risk for lead were identified by ranking census tracts, within each lead‐risk factor, from highest to lowest risk. Lastly, for simplicity smelter and manufacturing sites where lead smelting was also performed will be solely referred to as smelters for the remainder of the paper.

### 2.3 Data Collection

Data for the 13 considered lead‐risk factors (Table 1) were collected from various sources. Blood lead levels, demolition, and housing code violation data are collected by the City of Philadelphia and were retrieved from OpenDataPhilly (OpenDataPhilly, 2021). Lead‐risk factors such as children in poverty, age of property, median income, and minority population were collected by the U.S Census Bureau and taken from the 2015–2019 American Community Survey (U.S. Census Bureau, 2020). Because the American Community Survey only provides housing data by 10‐year increments, this research will be evaluating homes built before 1980, instead of 1978, to assess the likely presence of lead paint. The soil data were collected by the U.S. Environmental Protection Agency (U.S. EPA) and through the University of Pennsylvania's Academically Based Community Service (ABCS) Course on Lead and by graduate and undergraduate students working through The Community Engagement Core of Penn Medicine's Center of Excellence in Environmental Toxicology (CEET). Many of the soil samples were collected by residents, as organized by the EPA, the ATSDR, universities, and farmer/gardener groups, who generally collected five samples from the top 0–15 cm of soil in their yards, and recorded the nearest intersection for the sake of identity protection. Lead concentrations were then determined using portable X‐ray fluorescence (XRF) spectrometers which included: Innov‐X 4000 SL; NITON XLt792 YW; Innov‐X Delta; Olympus Delta Professional with 40 kV Tube and SDD detector custom configured with modes for soil; Thermo Fisher Scientific XL 3t 600. In the case of the ABCS course and CEET sampling, soils were collected primarily by students between 2015 and 2020, where students were instructed to collect 5 samples from the top ∼1.5 cm of soil. Again, portable XRF spectrometers were used to determine the lead concentration. Smelter and manufacturing site data were retrieved from both the EPA's Superfund Program Database and the paper Discovering Unrecognized Lead‐Smelting Sites by Historical Methods (Eckel et al., 2001; U.S. EPA, 2016). Brownfield and land recycled sites were taken from the PA‐DEP website (PA‐DEP, 2021a, 2021b)

### 2.4 Mapping and Statistical Analysis

Maps produced in this analysis used the geographic entity codes and coordination values for Philadelphia census tracts as taken from the U.S. Census Bureau's (2020) Philadelphia census tract level shapefile. The lead‐risk factors were then mapped on top of the shapefiles using RStudio Version 1.3.1093 (R Core Team, 2013). White areas within the maps are census tracts that were omitted (e.g., 9800, 9801, 9805, and 9809) or had inconsistencies in labeling within the data set. Gray areas within the maps are census tracts where data were unavailable for the specific factor mapped. A statistical online resource, “Social Science Statistics”, was used for the Spearman correlation analysis within this study (Social Science Statistics, 2018). All correlations discussed in this research are statistically significant (*p* < 0.05). Statistically non‐significant correlations are available in the data repository (Caballero‐Gomez et al., 2021).

## 3 Results

### 3.1 Demographic Lead‐Risk Factors

Demographic lead‐risk factors are community characteristics that, when present within a region, indicate a higher likelihood that the community is at risk for lead poisoning. The demographic lead‐risk factors evaluated in this study are based on the CDC's designated at‐risk populations for childhood lead poisoning (CDC, 2019) and include low‐income households and communities of color, especially non‐Latine Black communities. In addition, although renter‐occupied units were not included as a lead‐risk factor for the census tract level lead‐risk assessment, we still investigated the renter‐ and owner‐occupied housing data, because according to the CDC (CDC, 2019), renter‐occupied housing units are typically regarded as a lead‐risk factor.

#### 3.1.1 Renter‐ and Owner‐Occupied Housing Units

**Figure 1:**
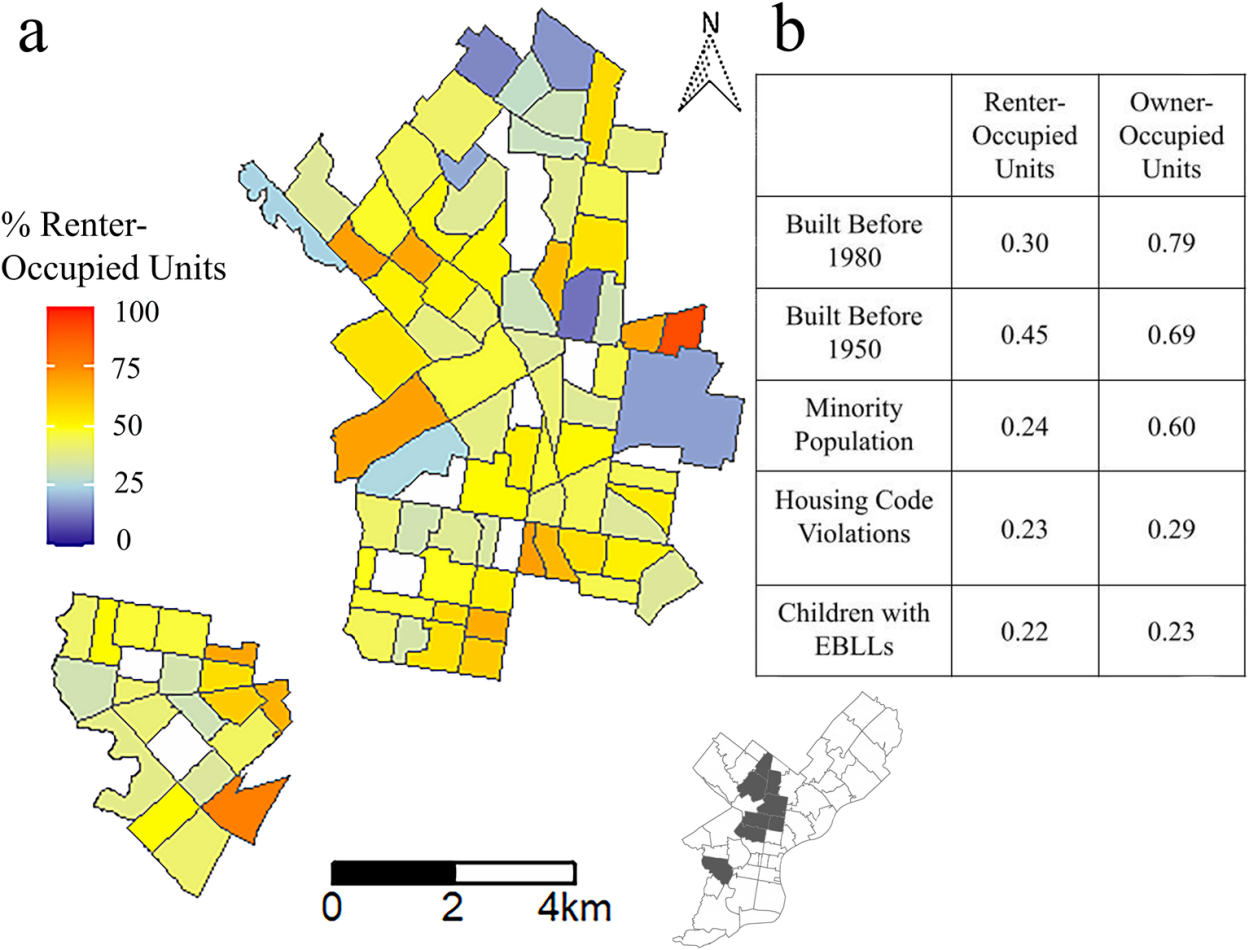
(a) Map illustrating the percentage of renter‐occupied units across the census tracts analyzed; (b) Comparison of Spearman correlation coefficients for renter‐occupied and owner‐occupied units across various lead‐risk factors. The inset shows a map of Philadelphia with all the high‐risk zip codes, that is, the region of focus in this study, highlighted in gray. Because the inset outlines zip codes rather than census tracts, the map and the inset do not perfectly align.

Approximately half of the census tracts (50/94) evaluated within this study had nearly equal representation of owner‐occupied and renter‐occupied housing units within their populations (Figure 1a). Sixteen of the evaluated census tracts (161, 171, 235, 248, 263.02, 264, 265, 266, 267, 270, 277, 280, 281, 288, 289, and 389) had more than half of housing units owner‐occupied (Figure S4 in the Supporting Information S1), whereas the remaining twenty‐eight census tracts (69, 77, 78, 79, 86.01, 139, 140, 147, 148, 153, 163, 164, 165, 166, 170, 173, 176.02, 199, 201.01, 206, 239, 240, 241, 242, 245, 246, 268, and 287) had more than half of housing units renter‐occupied.

#### 3.1.2 Black and Minority Population

A majority of the census tracts (76/94) evaluated were majority Black communities (Figure S5 in the Supporting Information S1). In two of the ninety‐four census tracts (67 and 77), the majority of the population belongs to a non‐Black minority (Figure S6 in the Supporting Information S1). The remaining sixteen census tracts (67, 77, 161, 162, 163, 164, 175, 176.01, 176.02, 195.01, 197, 198, 199, 206, 235, 236, 287, 288, 289, 383, and 389) have a white majority and are located along the border between North Philadelphia and the River Wards (see Figure S3 in the Supporting Information S1), a section of Philadelphia that is undergoing gentrification (Lipp, 2016; Lubrano & Gammage, 2019).

#### 3.1.3 Median Income

A majority of the census tracts (62/92) for which median income data were available are below the U.S poverty line, which is defined as a median family income of $26,200/year for a family of four (Figure S7 in the Supporting Information S1) (U.S. Census Bureau, 2021). Thirty‐one of those census tracts had median incomes below $20,000/year, and five of those census tracts (148, 151.01 164, 176.01, and 241) are considered in deep poverty, that is, with a median family income of $13,100/year for a family of four (U.S. Census Bureau, 2021). Only four census tracts (235, 236, 264, 270) have median incomes greater than $40,000/year, with two of these census tracts above $67,000/year (235 and 236). Three of the four census tracts with median incomes greater than $40,000/year are among the census tracts with more than half of housing units owner‐occupied (Figure S4 in the Supporting Information S1). The two census tracts with exceptionally high median incomes (235 and 236) also have a majority white population (Figure S6 in the Supporting Information S1).

### 3.2 Properties Built Before 1980 and 1950

A majority (61/94) of the census tracts evaluated have 90% of housing units built before 1980 (Figure S8 in the Supporting Information S1). In two of the census tracts (248 and 264), 100% of housing units were built before 1980. Within these 61 census tracts, there are 113,597 pre‐1980 housing units, which thus could pose a risk for lead hazards. Only four census tracts have less than 63% of housing units built before 1980, and these neighboring tracts are located along the border between North Philadelphia and Lower North Philadelphia.

Of the census tracts studied, only two census tracts (389 and 289) had more than 30% of housing units built before 1950 (Figure S9 in the Supporting Information S1). Fourteen census tracts (69, 70, 171, 200, 263.02, 264, 265, 276, 280, 281, 282, 288, 289, and 389) had at least 20% of housing units built before 1950.

### 3.3 Housing Code Violations

Between 2007 and 2020, the City of Philadelphia reported 499,435 housing code violations across the census tracts analyzed in this study. A majority of these census tracts are clustered in North Philadelphia (Figures S10 and S11 in the Supporting Information S1), primarily the Strawberry Mansion area (see Figure S3 in the Supporting Information S1). Of the housing code violations reported, 6085 (1%) are critical housing code violations (Figure S12 in the Supporting Information S1). Twenty‐eight of these 6085 critical housing code violations, spread across 21 census tracts (65, 72, 74, 80, 82, 83, 164, 165, 169, 175, 176, 197, 198, 199, 200, 201, 240, 243, 282, 283, and 284; Figure S12 in the Supporting Information S1), are lead‐hazard violations. Licenses and Inspections (L&I), Philadelphia's department responsible for the safety and livability of housing units, has had a long history of poor regulation and inefficiency due to chronic understaffing and inadequate funding to meet public demand for essential services (Howell & Lazzara, 2014; Randall, 2019). One survey of Philadelphia tenants and landlords even indicated there were loopholes to building and development regulations that allow landlords to evade Philadelphia housing standards (Howell & Lazzara, 2014). For this reason, lead‐hazard violations are likely underrepresented. Sixteen census tracts (71, 74, 137, 149, 151, 152, 164, 165, 167, 168, 169, 172, 175, 201, 265) had over 100 critical housing code violations (Figure S12 in the Supporting Information S1), whereas seven (71, 137, 152, 161, 169.01, 169.02, and 172) had more than 10,000 housing code violations within the 13‐year time period reported.

### 3.4 Demolitions

Between 2007 and 2020, the City of Philadelphia reported 5233 demolitions within the region studied in this research, and 3792 (73%) of those demolitions were due to housing code violations. Of the 94 census tracts analyzed, only one census tract (267) had no demolitions within the timeframe. Sixteen census tracts (169.02, 167.01, 169.01, 168, 137, 165, 149, 152, 172.01, 138, 161, 153, 175, 164, 174, and 151.01) had 100 or more demolitions (Figure S13 in the Supporting Information S1). Three of the census tracts (169.02, 167.01, and 169.01) had over 200 demolitions, with 90% of the demolitions as a result of housing code violations; these three tracts are geographically located alongside each other in the Strawberry Mansion area (Figure S14 in the Supporting Information S1).

### 3.5 Historic Smelters

Of the 42 historic smelters known to date within Philadelphia, six are in the region of study (Eckel et al., 2001; U.S. EPA, 2016; West Chester University, 2015). The six smelters are within 5 census tracts (139, 152, 161, 173, and 241), with census tract 161 having two smelters. Four of the historic smelters are secondary smelters: A. Perez & Son Division of Abrams Metal Co. Lead Smelter; Jos. Rosenthal's Sons Smelter; American Alloys Co.; and Electric Storage Battery. The other two, an unnamed smelter within census tract 161 and American Alloys Co., were likely primary smelters, although not confirmed in the smelter source materials (Eckel et al., 2001; U.S. EPA, 2016).

### 3.6 Soil Samples

Within the region of interest, there are 570 lead‐in‐soil data points documented (Figures S15 and S16 in the Supporting Information S1), of which 155 (27%) soil‐sampling points record hazardous soil lead levels, as defined by the EPA for bare residential soils where children play, that is, with lead contents of 400 ppm or greater (Rieuwerts & Farago, 1995). Some soil‐sampling points exhibit extremely high lead contents, with values ranging from 1000 to 5341 ppm. A majority of the lead‐in‐soil data are concentrated within 10 census tracts (71, 72, 73, 74, 78, 79, 80, 202, 239, and 244) in West Philadelphia, where a total of 381 (67%) data points are located (Figure S15 in the Supporting Information S1). For census tract 74 alone, there are 86 soil data points. For 34 of the 94 census tracts (36%), there are no soil data available at all, indicating a significant gap within the data set (Figure S16 in the Supporting Information S1).

### 3.7 Brownfield and Land Recycled Sites

Within the region of study, there were no brownfield sites documented. However, there were 23 land recycled sites within 20 census tracts (71.01, 73, 74, 78, 80, 137.01, 139, 148, 161, 163, 165, 166, 169.01, 170, 171, 175, 176.01, 203, 240, and 287; Figure S17 in the Supporting Information S1). These sites were cleaned up between 2003 and 2020. In nine of the census tracts, the contaminant identified was leaded gasoline, indicating residuals from leaded gasoline as a lead‐hazard source within Philadelphia. The remaining fourteen land recycled sites had contaminants generically labeled as “lead contaminants”.

Twenty‐one of the 23 land recycled sites had lead‐contaminated soil, eight of those sites had lead‐contaminated groundwater, whereas two sites solely had lead‐contaminated groundwater. A majority of the land recycled sites are located in census tracts with none to very low concentrations of hazardous soil lead levels (Figure S18 in the Supporting Information S1). In three of the land recycled sites (78, 166, and 175), activities on and use of the sites were limited due to severe contamination even after the clean‐up.

### 3.8 Elevated Blood Lead Levels

In 2015, a total of 70,750 children were screened for lead in blood within the census tracts analyzed here. Of the screened children, 4059 (6%) were reported to have EBLLs, that is, a BLL ≥5 μg/dL. However, the number of children screened ranged greatly among the studied census tracts, with some census tracts reporting only 30 children screened, whereas others reported 685. Sixteen census tracts (77, 86, 148, 153, 162, 166, 200, 204, 205, 206, 241, 248, 264, 269, 270, 287, and 389) screened less than 50% of children 0–5 years old. The percentage of screened children with EBLLs ranges among census tracts from 3.4% to 17.6%, with census tract 200 having the highest percentage. Thirty‐one census tracts (200, 283, 204, 169.01, 166, 203, 169.02, 151.01, 202, 238, 167.01, 149, 245, 201.01, 172.01, 247, 242, 173, 172.02, 284, 171, 168, 74, 244, 174, 246, 72, 138, 252, 282, and 281) had 10% or more children screened with EBLLs (Figure S19 in the Supporting Information S1).

### 3.9 Census Tracts With the Highest Lead Risk

The census tracts with the highest evidence of lead risk are 175 and 172, which are also environmental justice areas. These two census tracts were ranked high risk in eight of the thirteen lead‐risk factors evaluated (Figure S20 in the Supporting Information S1). It is important to note that the census tracts most frequently appearing as high risk are concentrated within the Strawberry Mansion area (Figure S3 in the Supporting Information S1), especially if including the census tracts considered high risk in seven of the 13 lead risk factor variables (Figure S20 in the Supporting Information S1).

## 4 Discussion

### 4.1 Risks of Lead Poisoning Associated With Owner‐Occupied Housing

The CDC states that a high percentage of renter‐occupied units within a community is a lead‐risk indicator (CDC, 2019). This relationship exists because rental units, especially low‐income housing, tend to have more health code and housing code violations than owner‐occupied homes (Howell & Lazzara, 2014; Krieger & Higgins, 2002; Randall, 2019). This issue can be compounded by a lack of sociopolitical capital within low‐income neighborhoods (Chisholm et al., 2018). In comparison, homeowners, in most cities, are in a higher income class and therefore are monetarily in a better position to preserve their homes.

Philadelphia, however, has had a historically high homeownership rate due to housing policy in the nineteenth and early twentieth centuries, which promoted the building of affordable rowhomes (Randall, 2019). This policy resulted in a large mass of old housing stock so that 88% of housing in Philadelphia was built before 1980. The impact of this housing policy is visible in Figure 1a, where in many census tracts, approximately half of the housing units are owner‐occupied, despite only four census tracts having median incomes greater than $40,000/year (Figure S7 in the Supporting Information S1). Significantly, eight of the fourteen census tracts (171, 263.02, 265, 280, 281, 288, 289, and 389) with at least 20% of housing built before 1950 (Figure S9 in the Supporting Information S1) also coincide with the 16 census tracts with more than 50% of the housing units owner‐occupied (Figure S4 in the Supporting Information S1). These findings suggest a high risk of lead pipes within owner‐occupied housing units. Owner‐occupied housing and properties built before 1980 show the highest correlation among the lead‐risk factors evaluated in this research (*R* = 0.79; Figure 1b and Table S2 in the Supporting Information S1) and present a nearly identical risk of EBLLs in contrast to what has previously been reported in other cities. This result documents a unique housing lead‐risk situation in Philadelphia.

This unique situation is most evident when comparing the correlation values between renter‐occupied and owner‐occupied housing units to lead‐risk factors (Figure 1b and Table S2 in the Supporting Information S1). Census tracts with a high percentage of owner‐occupied units have higher likelihoods of also having a higher percentage of pre‐1950 buildings and minority populations than census tracts with many renter‐occupied units (Figure 1). Older buildings and minority populations are both lead‐risk factors established by the CDC, which are typically associated with renter‐occupied housing (CDC, 2019). There is also a slightly higher correlation between owner‐occupied units and other lead‐risk factors, such as housing code violations, than there are with renter‐occupied units (Figure 1b).

Housing code violations within areas with a high percentage of owner‐occupied units are likely underrepresented since the City of Philadelphia requires rental properties to be inspected for a rental property license, but this level of regulation and inspection does not exist for owner‐occupied housing. Based on these results, Philadelphia's lead policy and support should be reimagined to increase both attention and resources to homeowners alongside current efforts to support renters. Specifically, census tracts 171, 263.02, 265, 280, 281, 288, 289, and 389 should be further supported as they simultaneously have a larger homeowner population and a higher number of old housing units.

### 4.2 Demolitions as a Critical Risk Factor for Childhood Lead Poisoning

**Figure 2:**
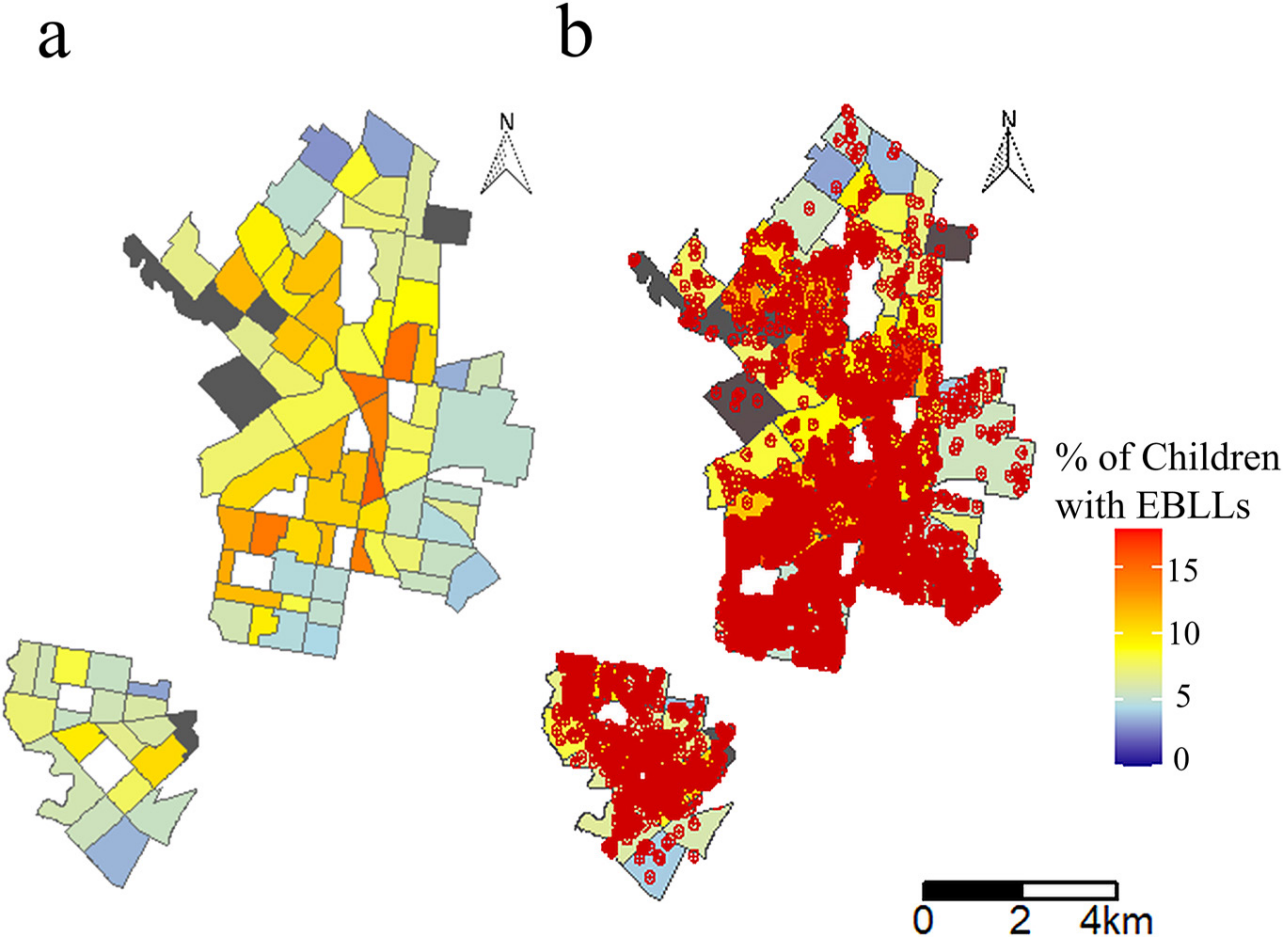
(a) Map presenting the percent of children with EBLLs by census tract, as collected in 2015 by the City of Philadelphia; (b) Map displaying each housing unit demolished (red dot) from 2007 to 2020 and each demolished unit's 122‐m radius of impact (red circle) superimposed onto the EBLL map shown in (a). The EBLL legend applies to both maps.

Demolitions affect mostly buildings constructed before 1980 which likely contain lead paint. Therefore, demolitions are a risk indicator of EBLLs in children, likely due to the release of lead‐contaminated dust from paint (Figure 2). Lead‐contaminated dust from demolitions that has settled on streets, that is, road dust, or soil can be tracked or blown into nearby homes, posing a threat for the residents, especially children (Farfel et al., 2003; Rabito et al., 2007). Therefore, demolition sites and their radius of impact, defined as the 122‐m radial range where risk of lead exposure due to demolitions is possible (e.g., Lauer, 2019), were mapped alongside EBLLs. Our analysis revealed that demolition points and radius of impact overlap in part with census tracts where many children have EBLLs, marked by the orange colors (Figure 2). It is of note that there exist some areas with a low percentage of children with EBLLs yet a high number of demolitions, but the association between demolitions and EBLLs is further validated by the relatively strong correlation between demolitions and the percent of children with EBLLs (*R* = 0.55; Table S2 in the Supporting Information S1) and more clearly visible when demolitions are superimposed onto a map displaying the absolute number of children with EBLLs (Figure S21 in the Supporting Information S1). In addition, census tracts, within Figure 2, with a low percentage of children with EBLLs yet a high number of demolitions also overlap with census tracts that have the lowest percentage of properties built before 1980 (Figure S8 in the Supporting Information S1) and have fewer housing code violations (Figure S10 in the Supporting Information S1) compared to those with a high percentage of children with EBLLs and a high number of demolitions. Our result is critical considering previous studies found that exposure to multiple demolitions within a residential block was associated with a significant increase in BLLs within children and with cumulative increases in inhalation of ambient lead (Farfel et al., 2003; Rabito et al., 2007). Thus, our study in combination with previous evidence (Figure 2), supports a clear link between EBLLs and the demolition of old buildings, especially in North Philadelphia (Figure S22 in the Supporting Information S1). The data, therefore, establish that the demolitions of buildings built prior to 1980 within West and North Philadelphia are a critical risk factor for childhood lead poisoning.

Given the findings in this investigation, demolitions in Philadelphia need further regulation to minimize the risk of lead‐contaminated dust exposure. To be effective, however, such regulations must be enforced, which requires inspections for compliance. The “East Baltimore Protocol” is a strict protocol for demolition, which mandates, among other procedures, wetting the structure before and during demolition, along with wetting debris before covering and transporting it. This protocol was found to generate 56 times less lead‐contaminated dust in nearby areas during demolition as well as during debris removal, due to stricter regulation compared to dry demolition (Lauer, 2019). Philadelphia's demolition standard, however, does not require wetting of the structure before or during demolition, but merely recommends applying water or an approved dust suppressant to minimize dust. These recommendations at most reduce lead‐contaminated dust production by 2.6 times (Lauer, 2019). The City of Philadelphia should therefore make demolition requirements more stringent, for example, by applying those contained in the “East Baltimore Protocol”. Following this protocol is more time‐consuming and therefore more costly. However, its significant reduction in lead‐contaminated dust generation, combined with the evidence from our study, demonstrates its necessity in Philadelphia. Nevertheless, even when following the “East Baltimore Protocol”, the danger of lead‐contaminated dust is present. For this reason, it is important to prioritize the maintenance and rehabilitation of buildings within Philadelphia, instead of demolition. It has been shown that rehabilitation and preservation of buildings improve economic, social, and emotional outcomes of residents, whereas demolitions often accelerate gentrification and the displacement of marginalized communities (Randall, 2019). Further analysis should compare the lead risk associated with the East Baltimore Protocol versus rehabilitation of structures for those residents who continue to live within the building and surrounding area.

### 4.3 Lead Paint and Demolitions Are Critical Lead‐Source Emissions for Childhood Lead Poisoning

**Figure 3:**
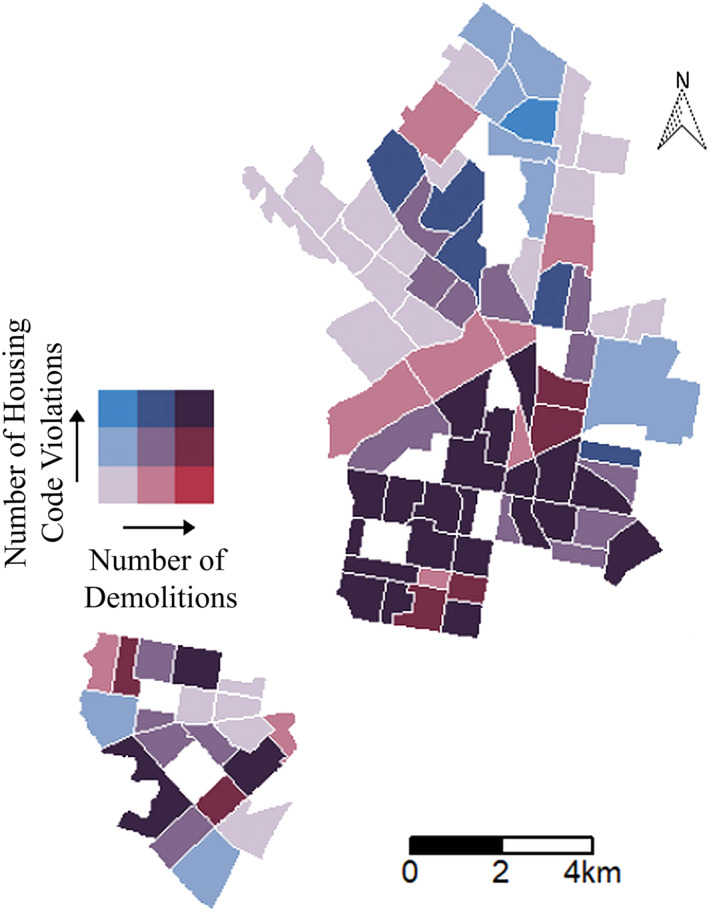
A bivariate map that presents census tracts where demolitions and housing code violations are the most prevalent, illustrated by the deep purple color. Census tracts with a high number of demolitions are represented by increasingly darker pinks, whereas census tracts with a high number of housing code violations are represented by increasingly darker blues. Census tracts with few demolitions and housing code violations are represented by a light lavender.

Housing code violations and demolitions were found to be correlated in this study (*R* = 0.74, Table S2 in the Supporting Information S1). Considering that housing code violations are associated with paint deterioration and the existence of other lead‐paint hazards, a higher number of housing code violations increases the likelihood of lead hazards present in a home (HUD, 2002). Since housing code violations also correlate with demolitions, it is likely that demolitions in Philadelphia are releasing and redistributing lead‐contaminated dust. Lead‐contaminated dust accumulation on ceilings and roofs is especially relevant in older and poorly maintained housing as lead‐contaminated particles have settled during earlier, more industrial periods and were able to enter and remain within cracks and crevices of the structures (Davis & Gulson, 2005). Therefore, a high number of housing code violations would indicate a higher risk of lead‐contaminated dust from ceilings and roofs. The high correlation value between census tracts with a high percentage of children who have EBLLs and census tracts with a high number of housing code violations (*R* = 0.68; Figure S23 and S24 in the Supporting Information S1) corroborates the conclusion that demolition of units with housing code violations produce lead‐contaminated dust hazards. Based on the correlation values there appears to be a compelling relationship between housing code violations, demolitions, and the number of children with EBLLs. This relationship strongly suggests that a major cause of EBLLs in West and North Philadelphia is the ingestion of lead‐paint dust and other forms of lead‐contaminated dust in housing, in agreement with City reports and prior studies (Hauptman et al., 2017; Philadelphia Department of Public Health, 2018; Sergio & Sordo, 2006). Therefore, these two lead‐risk factors are shown together in Figures 3 and S25 to identify the areas of highest lead risk due to lead paint and dust. Census tracts with the highest lead risk due to lead paint deterioration and dust are: 65, 74, 85, 137, 138, 140, 149, 151.01, 152, 153, 164, 165, 167.01, 168, 169.01, 169.02, 173, 174, 175, 201.01, 202 (deep purple in Figure 3). Many of these census tracts border each other, creating a large section in North Philadelphia that is at high risk of lead poisoning due to contaminated dust from lead paint and other sources, such as soil (Bradham et al., 2017). Given that contaminated dusts from lead paint and other sources, such as soil, are regarded as a major source of childhood lead poisoning, housing within these census tracts, and especially this section of North Philadelphia, needs to be investigated for lead hazards and remediated as soon as possible to decrease childhood lead poisoning (see also Rabito et al., 2007; Sergio & Sordo, 2006). This would require redirection of funding, greater staffing and more stringent regulation efforts on the part of L&I to inspect renter‐occupied and in addition owner‐occupied housing (HUD, 2002).

### 4.4 Low‐Income Housing is at Greater Risk for Housing Code Violations and Demolitions

Median income is negatively correlated with demolitions (*R* = −0.65, Table S2 in the Supporting Information S1). This correlation suggests that lower‐income communities are more likely to be at risk of lead exposure through the demolition of buildings. Our finding is in line with demolition practices generally, as substandard housing within U.S. cities is demolished at higher rates than other housing units (Rabito et al., 2007). Within Philadelphia's Department of L&I, the demolition of housing units with hazards is standard practice; due to a lack of funding, when a housing unit exhibits hazards, L&I tends to demolish the building instead of repairing it, because, if at a later date the building is responsible for an injury and/or health impact, the liability would be costly (Randall, 2019). Median income is also negatively correlated with demolitions due to housing code violations (*R* = −0.65; Table S2 in the Supporting Information S1). These results suggest that demolitions occur at higher rates within low‐income communities because units are more likely to have housing code violations. Therefore, our results indicate that low‐income housing is more likely to have housing code violations, which are associated with increased risk of childhood lead poisoning, and result in demolitions, which in turn elevate the risk of lead poisoning. This is an example of environmental injustice where low‐income communities of color experience the disparity of elevated lead exposure in part due to L&I practice of enhanced demolition.

### 4.5 Racial Disparities in Housing Standards and Risk of Lead Exposure

Predominantly Black communities are correlated with EBLLs, housing code violations, and properties built before both 1980 and 1950 (*R* = 0.53, 0.50, 0.66, and 0.54, respectively; see Table S2 and Figures S26, S27, S28, and S29 in the Supporting Information S1). These findings point to significant racial disparities in housing standards and risk of exposure to lead. Our results indicate that Black children are at the highest risk for lead poisoning within West and North Philadelphia. These results are consistent with the CDC's conclusion that non‐Latine Black children and communities are at highest risk for lead poisoning and the associated social impacts (CDC, 2019). For this reason, greater support needs to be provided for schools and health facilities in predominantly Black neighborhoods within Philadelphia. These efforts should be done in addition to primary prevention of lead poisoning by providing financial support toward household rehabilitation and inspection for low‐income and lower‐middle‐class Black families in Philadelphia.

### 4.6 Potential Relationship Between Historical Smelters and Childhood Lead Poisoning

Due to poor regulation, lack of health‐informed policy, and economic interests, the emissions from U.S. smelters were not monitored or restricted in the early nineteen hundreds, limiting our ability to evaluate past deposition and present lead impact due to lead's persistence in soil from smelter fallout (Sullivan, 2015). Smelter emissions can travel large distances from the site of smelting, creating a significant radius of impact, which must be considered when evaluating soil lead levels near smelter sites (Rieuwerts & Farago, 1995). Based on a literature review that compiled soil studies around smelters, the greatest distance from a smelter with hazardous soil lead levels, as defined by the EPA at 400 ppm, is 500 m (Rieuwerts & Farago, 1995).

**Figure 4:**
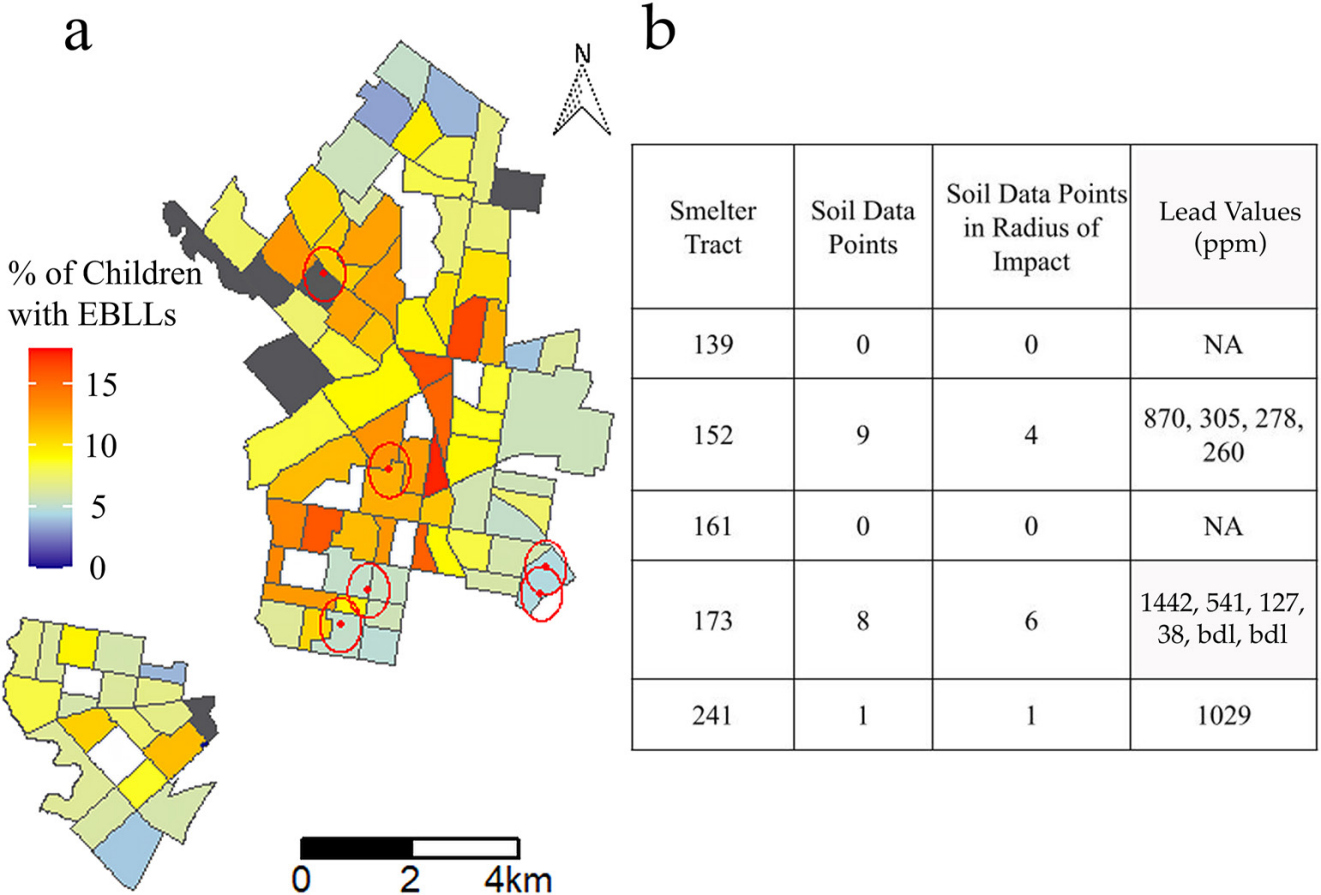
(a) Map displaying the sites of the six historic smelters in the study area (red dots) and each site's 500‐m radius of impact (red circles); (b) soil data for each census tract that hosts a former smelter, along with sampling points specifically within the smelter's radius of impact. bdl = below detection limit.

Historic smelter sites along with their 500‐m radius of impact were superimposed onto a map displaying Philadelphia's 2015 reported data on children's EBLLs by census tract (Figure 4a) (OpenDataPhilly, 2021). Since there are only six smelters in the studied area and only limited Pb‐in‐soil data are available for these locations, it is difficult to reach sound conclusions regarding the contribution of lead‐in‐soil from smelters. The available data for the soils close to these smelters, however, suggest that further data should be collected. From the data available we could not identify a relationship between smelters and children's EBLLs within the census tracts analyzed. Only two smelters have a radius of impact that seems to overlap with census tracts in which more than 10% of children have EBLLs. This may mean that historical smelters are not as critical a lead‐risk factor to consider when evaluating areas of high lead risk in Philadelphia, but additional data are required to support this preliminary conclusion. Notably, the majority of the 42 known former smelters in Philadelphia were located in the Riverwards (see Figure S3 in the Supporting Information S1) rather than in North and West Philadelphia (see O’Shea et al., 2021). Despite the paucity of lead‐in‐soil data points, hazardous soil lead levels were observed within the smelters' radius of impact in each of the smelter‐hosting census tracts, for which soil data were available (Figure 4b).

None of the brownfield and land recycled sites in the Philadelphia areas studied here are co‐located with historical smelter locations, although three sites do appear within the radii of impact. Based on this finding, it seems that these smelter sites have not been remediated and are likely to have lead contamination, especially considering the immobility of lead within soil. Previous studies within the Kensington neighborhood of Philadelphia reported that soil lead levels were significantly higher near historical smelters than at Philadelphia locations where no smelters previously existed (Lusby et al., 2015). It may be that other, more local forms of remediation, which are not documented, have been completed, such as gardens; however, since this is unverified there is a need for greater sampling of soil within these smelter‐hosting census tracts and their radii of impact.

### 4.7 Increased Efforts to Sample Soil Are Needed in North Philadelphia

The emissions from lead sources evaluated in this study primarily settle on soil. Lead‐contaminated dust and paint may also settle on roads, porches, and inside homes, but these sites are often inaccessible for sampling. Bodies of water may also be a final sink for lead particulates, but they are not relevant in the area examined in our study. As such, soil becomes the most accessible and relevant environmental medium to investigate for the identification of sites with a high lead risk in need of preventive action. Previous studies have even used soil‐Pb data to predict spatial variation in BLLs (Mielke et al., 2016) and reported that the resuspension of lead‐in‐soil explains the spikes in BLLs during the summer season (Laidlaw et al., 2017; Zahran et al., 2013). Despite resounding evidence for the existence of a relationship between BLLs and soil‐Pb levels (Bradham et al., 2017; Laidlaw et al., 2017; Mielke et al., 2016; Zahran et al., 2013), we did not find a statistically significant relationship between EBLLs and lead‐in‐soil concentrations (*R* = −0.24393; *p* = 0.067; see data repository) within the region of study. However, a previous study within Philadelphia identified a significant positive relationship between BLLs and soil‐Pb levels (Bradham et al., 2017), suggesting that our research may not have included sufficient soil data. Therefore, our research revealed a need for greater future sampling of soils within the area of study. Within this area, some census tracts were more densely sampled than others (primarily within West Philadelphia), making it difficult to compare lead risk from soil between these regions of Philadelphia and its association with reported EBLLs. Despite the limited amount of soil data in most census tracts (Figure S15 in the Supporting Information S1), only 22 of the ninety‐four (23%) census tracts evaluated do not contain sites with lead‐in‐soil values ≥ 400 ppm. Some of the samples are at exceedingly hazardous soil lead levels; in the most extreme case, the soil lead levels are up to 13 times the EPA hazard threshold of 400 ppm (Figure S16 in the Supporting Information S1). Although the majority of the hazardous soil data points were within West Philadelphia, where most of the sampling occurred, there are a few outside of this part of town, which suggests that more samples need to be collected from North Philadelphia.

Furthermore, recognizing that a child's EBLL is a reflection of recent cumulative exposure to lead from multiple sources, lead‐in‐soil data are a useful lead‐risk indicator for one important source of exposure in need of preventative action (Laidlaw et al., 2017). Therefore, any effort to effectively reduce BLLs must include soil remediation. Also, despite the federal reference level for hazardous soils being at 400 ppm for bare soils in play areas, various states have lowered the lead‐in‐soil threshold in response to concerns that the 400 ppm value was established through a risk assessment process using 10 μg/dL, the then current blood lead level that the CDC considered safe. Since 2012, the CDC has considered no amount of lead in blood to be safe, and since last year, has lowered the blood lead reference level to 3.5 μg/dL (CDC, 2021a, 2021b). The EPA has not yet incorporated this important understanding of low‐level lead risk into its threshold for lead‐in‐soil.

Evidence of historic smelters and significant numbers of demolitions with associated emission of lead‐contaminated dust in North Philadelphia further highlight a great need for soil sampling in this area. This is especially pressing when considering that previous work in Philadelphia identified floor dust at household entryways as a critical risk factor for childhood lead poisoning (Dignam et al., 2019). A few significant open‐land areas, which existed as green space when the smelters were likely active and continue to be green spaces with no evidence of remediation, include: a large public park and church within the radius of impact of the Cadman, A.W. MFG. CO. 2 Lead Smelter in census tract 241 (Figure S30 in the Supporting Information S1); several schools within the radius of impact of the A. Perez & Son Division of Abrams Metal Co. Lead Smelter, American Alloys Co. and another unnamed smelter in census tracts 139 and 161 (Figures S30 and S31 in the Supporting Information S1); and significant open‐land in the form of residential lawns within census tract 139 (Figure S30 in the Supporting Information S1), which being private property are less likely to be sampled and remediated (Mielke et al., 2007). Open‐land areas around smelters are especially important for remediation, as previous research has found remediating soil proximate to smelters with lead concentration >500 ppm resulted in a 2.5 μg/dL reduction in BLLs among children 3 years old and younger (Lanphear et al., 2003).

### 4.8 Limitations and Future Directions

There are several limitations to this study, however, including missing lead sources such as census tract‐level data on highly frequented roads, along which leaded gasoline emissions from mobile sources would have accumulated, and locations of existing lead service lines. The PA‐DEP's Land Recycling Program reports include only those Brownfield sites that have been identified for clean‐up so that they can be re‐used. There are likely numerous other contaminant sites that are not included in these reports because they are not commercially viable. There is also a gap in the availability of soil data within North Philadelphia that needs to be closed in order to better understand the risk within that region. Certain data are also highly underrepresented due to limitations within L&I, including data on housing code violations, and especially lead violations. For these reasons, this study can only present conclusions based on the available data, and therefore does not provide a complete representation of the cumulative lead risk within the studied area of Philadelphia. Moreover, despite this study investigating lead risk in Philadelphia at the smallest geographic unit done to date, it is limited by the representation of data at the census tract level. In one study, census blocks rather than census tracts were found to better predict BLLs and other health effects, as health outcomes are more closely associated with an individual's immediate environment than with larger geographic areas (Kaplowitz et al., 2010). This emphasizes the importance of further work identifying lead risk, along with other health risks, at a neighborhood level. Remediation and prevention efforts should also take into account the considerable role of the proximate environment on lead risk and health outcomes. In previous studies, significant decreases in lead contamination and BLLs were reported at the neighborhood level when remediation efforts targeted both high‐risk homes and surrounding homes (Laidlaw et al., 2017; Schoof et al., 2016).

Another point illuminated by the data used here was the disparity in BLL screening of children. Despite having the greatest proportion of children with EBLLs relative to the total number of children screened, census tract 200 is one of the 16 census tracts with less than 50% of children screened, and it does not emerge as one of the highest lead‐risk census tracts (Figure S20 in the Supporting Information S1). This result may indicate a need to increase screening across all census tracts, or point to a lead source that was not evaluated in this study but impacts census tract 200 and others in the region, for example, lead drinking water pipes or historic leaded gasoline emissions. Our research identified fourteen census tracts in which 25% of the housing units were built before 1950, including census tract 200. These census tracts should be prioritized for an investigation of lead pipes by the Philadelphia Water Department, followed by replacement of the identified lead service lines. Considering the CDC's recent decision to lower the blood lead reference value from 5 μg/dL to 3.5 μg/dL, significant efforts must be made to increase screening for children across Philadelphia, but especially in the identified high‐risk census tracts. These efforts would require increased access to on‐site testing, education across stakeholders (i.e., parents, physicians, local public health organizations), and greater collaboration with local health providers and community organizations. Efforts to increase both BLL screening and soil sampling can be done effectively and efficiently through community engagement, which improves community capacity and lowers the risk of adverse health outcomes (Bracic, 2018; O’Mara‐Eves et al., 2015). A reevaluation of lead‐risk factors among children with reported EBLLs using the new 3.5 μg/dL blood lead reference level would further improve our understanding of lead risk in Philadelphia.

A historical analysis could be used to investigate the legacy of leaded gasoline emissions in Philadelphia, which could be achieved by identifying and mapping roads that were heavily trafficked during the timeframe of leaded gasoline use, and by determining the lead isotopic composition of soils adjacent to these roads, which may carry a unique gasoline isotopic signature (see, for example, Wang et al., 2021), and thus would help in spatially analyzing the potential emission dispersion of gasoline‐derived lead. This information would help in further prioritizing remediation and preventative action. It is especially important to investigate leaded gasoline considering that nearly equal amounts of lead in gross‐tonnage were used in white lead pigment as was in leaded gasoline before the mandated decrease in their lead contents (Mielke & Reagan, 1998).

## 5 Conclusions

Lead paint, housing code violations, demolitions, and owner‐occupied housing continue to be lead‐risk factors in Philadelphia. A significant finding of our research is that the lead risk is highly correlated with owner‐occupied housing in Philadelphia due to high homeownership in low‐income areas. This association should be further investigated in other cities, as its implications for policy and prevention of lead poisoning are critical. Furthermore, this research identified that demolitions are strongly associated with EBLLs in children, pointing to lead‐contaminated dusts released during the demolition process as an important source, suggesting that dusts from lead paint play an important role in lead poisoning. Lead‐contaminated dust from demolition activity warrants additional regulatory mitigation and greater code enforcement, as it impacts low‐income communities of color, that is, environmental justice communities, considerably more than higher‐income white communities in Philadelphia. Census tracts 175 and 172, which were identified as having the highest risk for childhood lead poisoning, should have resources directed to them in order to increase efforts to remediate homes, test and support children within these areas. The 16 census tracts with less than 50% of children screened for EBLLs should receive targeted screening programs by the Philadelphia Department of Public Health.

It is important to note that the census tracts most frequently appearing as high risk are concentrated within the Strawberry Mansion area, especially when including the census tracts considered high risk in regard to seven of the thirteen lead‐risk factors (Figure S20 in the Supporting Information S1). Support to identified high‐risk communities should include holistic lead‐hazard screening and remediation for all lead sources (Schoof et al., 2016) as it is the most effective approach to decreasing childhood lead poisoning. The Federal Department of Housing and Urban Development uses an average per‐unit cost of $12,000 (HUD, 2018). Given the high costs of holistic screening and remediation, prioritization of high‐risk neighborhoods is the most financially feasible way of beginning to address the issue. This research consolidates lead risk at the census tract‐level for this purpose. In addition, this research identified open‐land space around former smelter sites that the EPA and the City should sample and remediate if necessary. Our results provide insights for public health policy moving forward, especially for the City of Philadelphia's Department of Public Health, Department of License's and Inspection, and City Council.

## Conflict of Interest

The authors declare no conflicts of interest relevant to this study.

## Supporting information

Supporting Information S1

## Data Availability

Datasets and script used to map data for this research are available at The Environmental Data Initiative (Caballero‐Gomez et al., 2021). The lead‐risk factor data used for the lead‐risk analysis and mapping in the study are all available at The Environmental Data Initiative which is released to the public domain under Creative Commons CCO 1.0. Version 1.3.1093 of RStudio was used for mapping data into visuals.
