# Supplementary material for: Spatial Analysis and Lead‐Risk Assessment of Philadelphia, USA
Source: Geohealth. 2022 Mar 1;6(3):e2021GH000519. doi: 10.1029/2021GH000519 (PMC8934574; doi:10.1029/2021GH000519)
Supplement: Supplementary file 1 — Supporting Information S1 [file GH2-6-e2021GH000519-s001.docx]

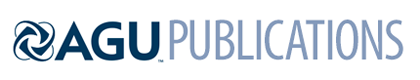


*GeoHealth*

Supporting Information for

**Spatial Analysis and Lead-risk Assessment of Philadelphia**

H. Caballero-Gómez^1^, H. K. White^1^, M. J. O’Shea^2^, R. Pepino^2,3^, M. Howarth^3^, and R. Gieré^2,3^

^1^Department of Chemistry, Haverford College, Haverford, PA 19041, USA

^2^Department of Earth and Environmental Science, University of Pennsylvania, Philadelphia, PA, 19104-6316, USA

^3^Center of Excellence in Environmental Toxicology, University of Pennsylvania, Philadelphia, PA, 19104-6316, USA

**Contents of this file**

Figures S1 to S34

Tables S1 to S2

**Additional Supporting Information (Files uploaded separately)**

Captions for Datasets S1

**Introduction**

The following supporting information includes maps and tables detailing the region of study, lead-risk factors by census tract, open land around smelter sites, and figures depicting current lead-in-soil data and the census tracts identified to be at the highest risk for childhood lead poisoning. Based on the statistical analysis, bivariate lead-risk factor maps were produced to depict high-risk census tracts by mapping strongly correlated lead-risk factors. The data for each map were collected as described in section 2.3 of the paper, and maps were created as described in section 2.4 of the paper. White areas within the maps are census tracts that were omitted (e.g., 9800, 9801, 9805, and 9809) or had inconsistencies in labeling within the data. Gray areas within the maps are census tracts where data were unavailable for the factor mapped.

**
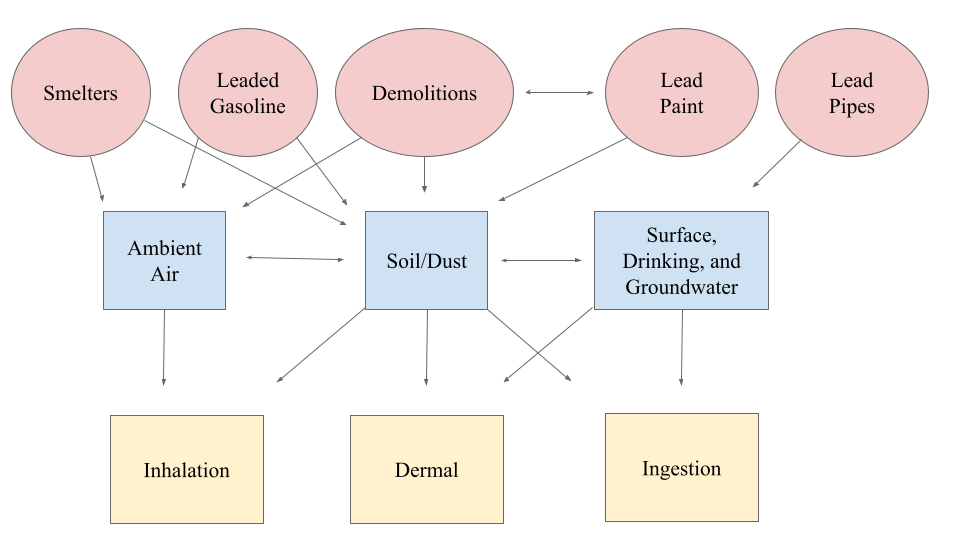
**

**Figure S1.** The five major sources of lead emissions in Philadelphia (red ellipses), along with the three environmental sinks (blue boxes) for lead emissions, and primary pathways of exposure (yellow rectangles) possible from each environmental sink.


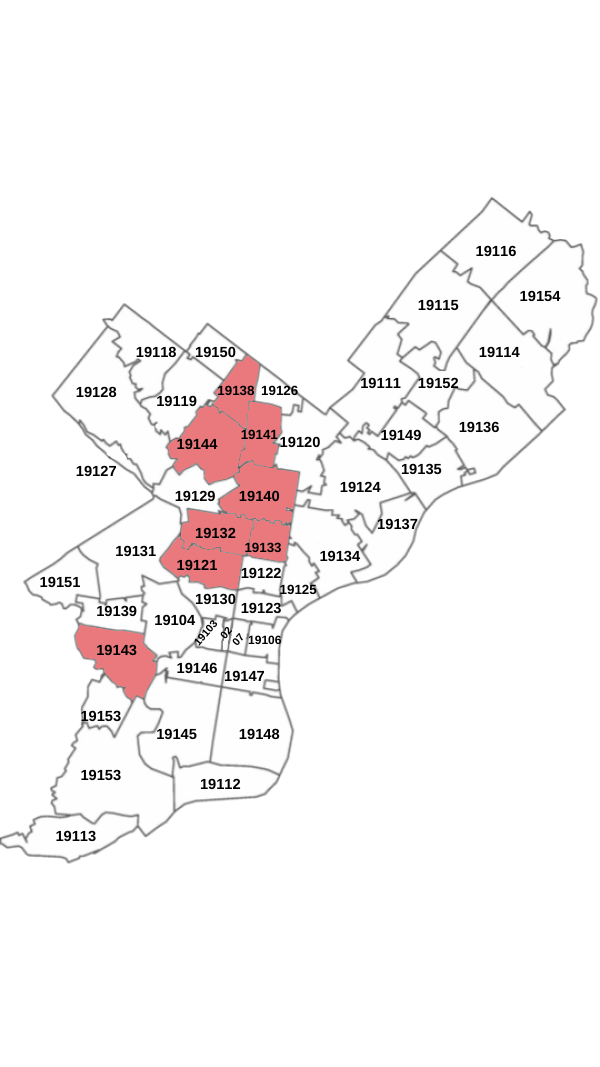


Figure S2. Map of Philadelphia zip codes with the studied high-risk zip codes, as identified by an initial lead-risk assessment, highlighted in red.


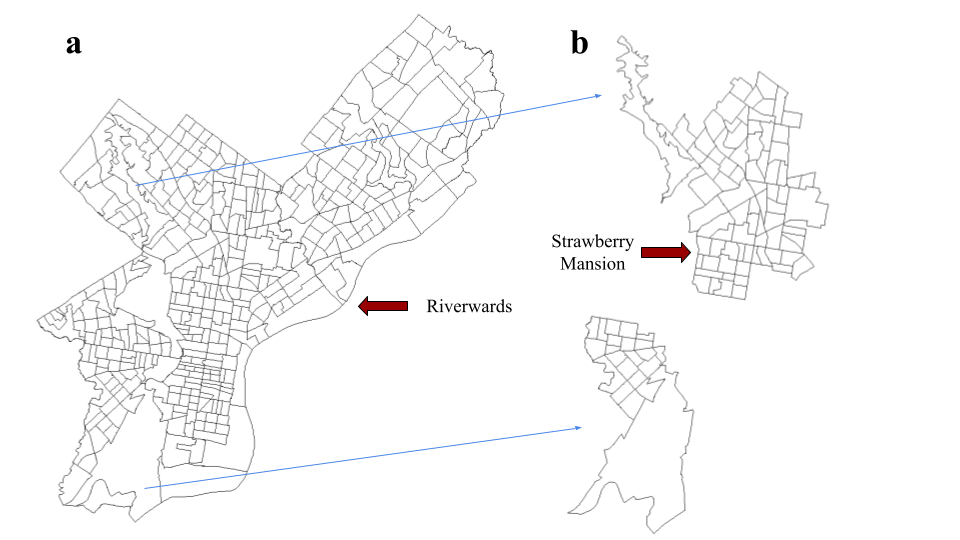


Figure S3. a) Map of Philadelphia census tracts, with a red arrow indicating where the Riverwards are located. b) Map of census tracts focused on within our study, with a red arrow pointing to the Strawberry Mansion area.

**
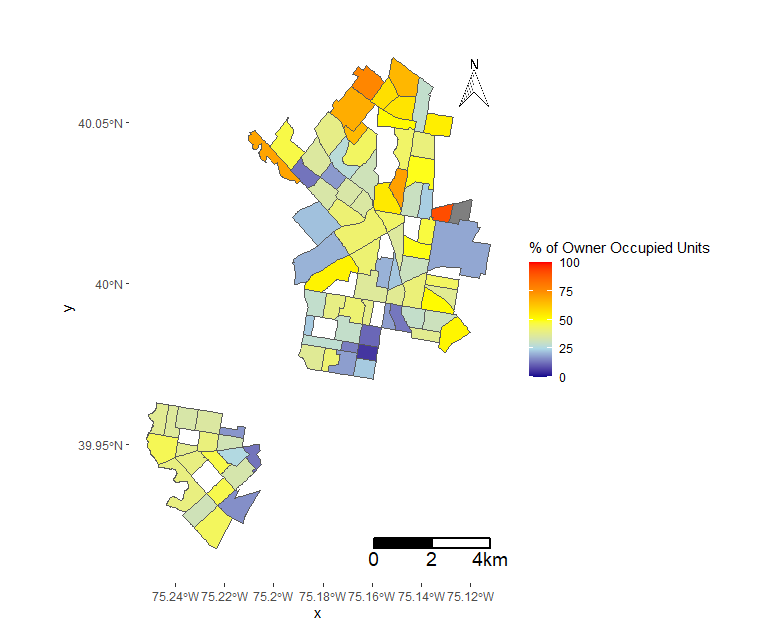
**

**Figure S4.** Owner-occupied units by census tract for the region of study.


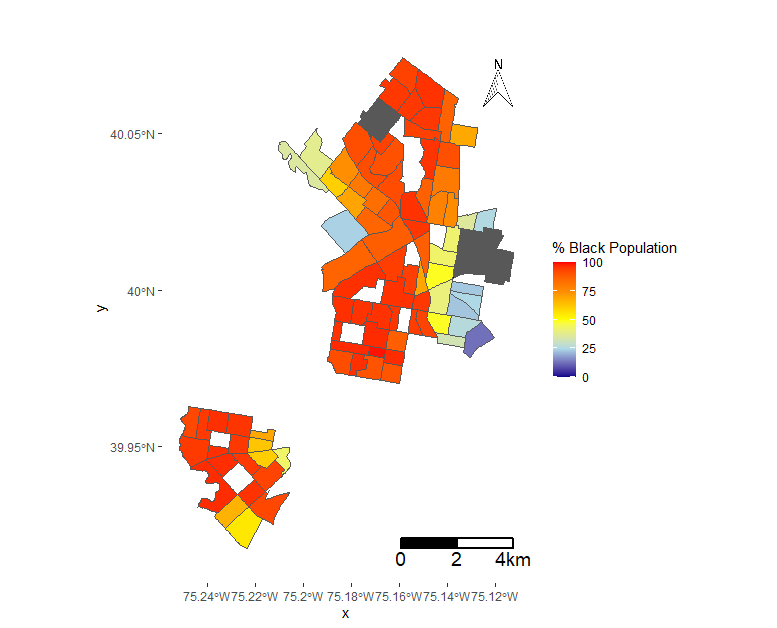


Figure S5. Black population by census tract for the region of study.


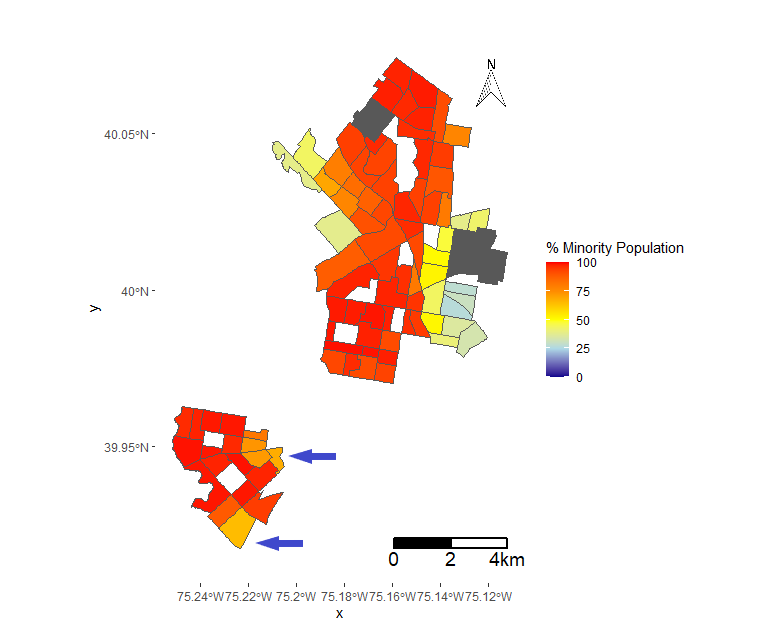


**Figure S6.** Minority population by census tract for the region of study. The blue arrows indicate census tracts with a non-Black minority majority.

**
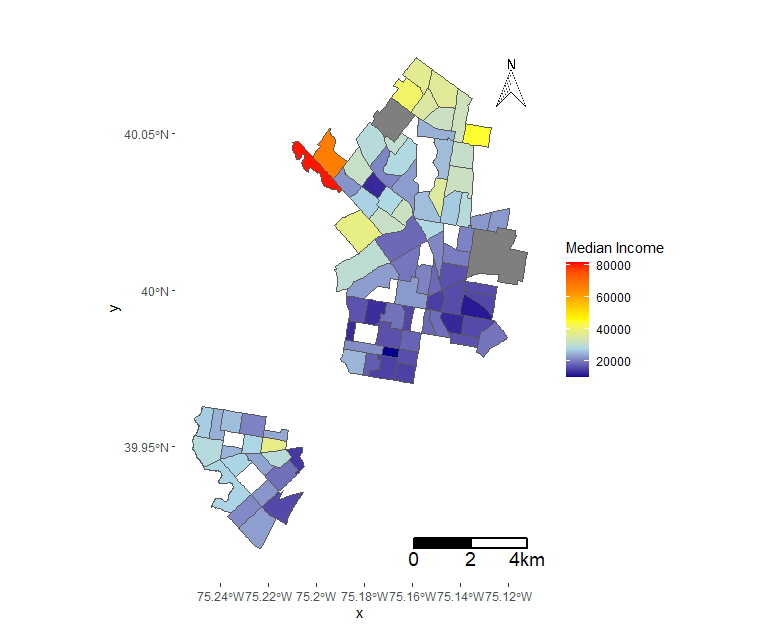
**

**Figure S7.** Median income by census tract for the region of study.

**
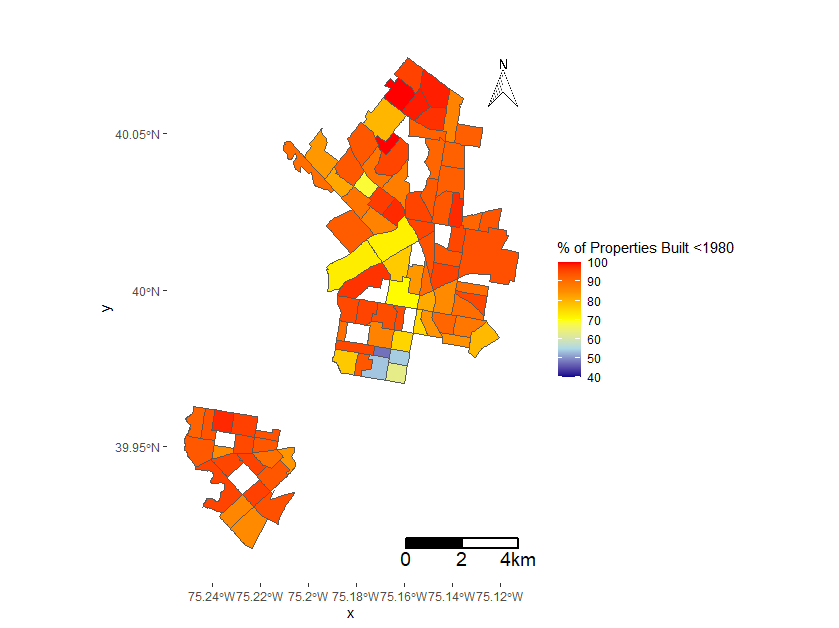
**

**Figure S8.** Properties Built Before 1980 by census tract for the region of study.

**
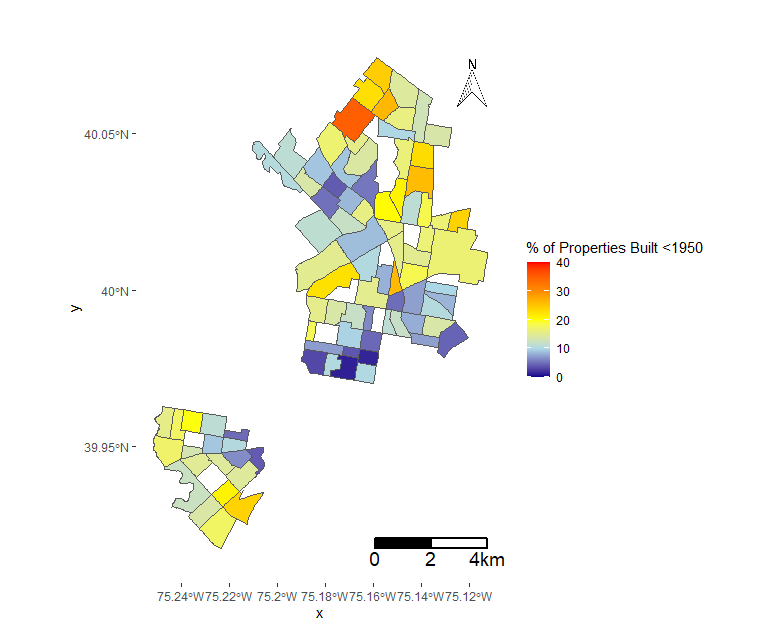
**

**Figure S9.** Properties Built Before 1950 by census tract for the region of study.

**
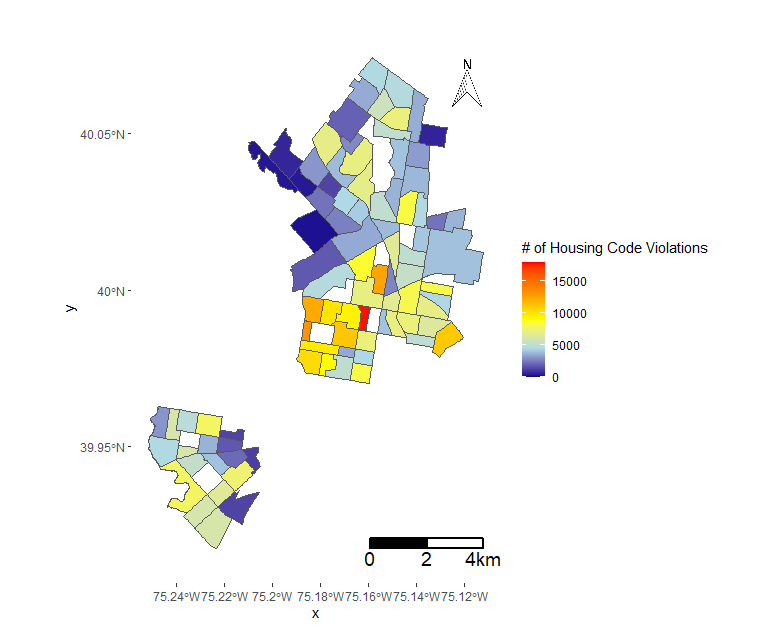
**

**Figure S10.** Housing code violations by census tract for the region of study.

**
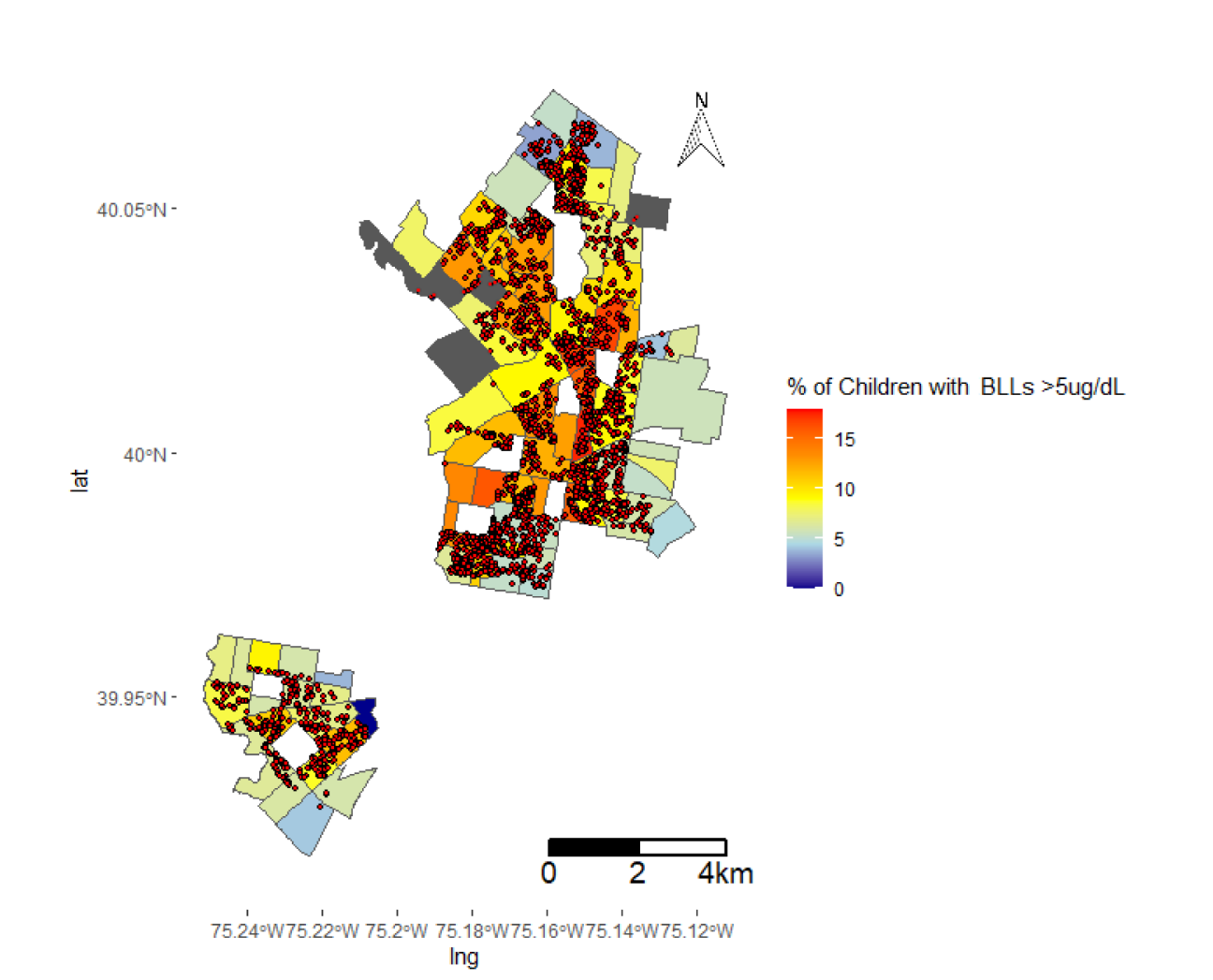
**

**Figure S11.** Housing code violations sites (dots) overlayed on the map showing the percentage of children with elevated blood lead levels by census tract for the region of study.

**
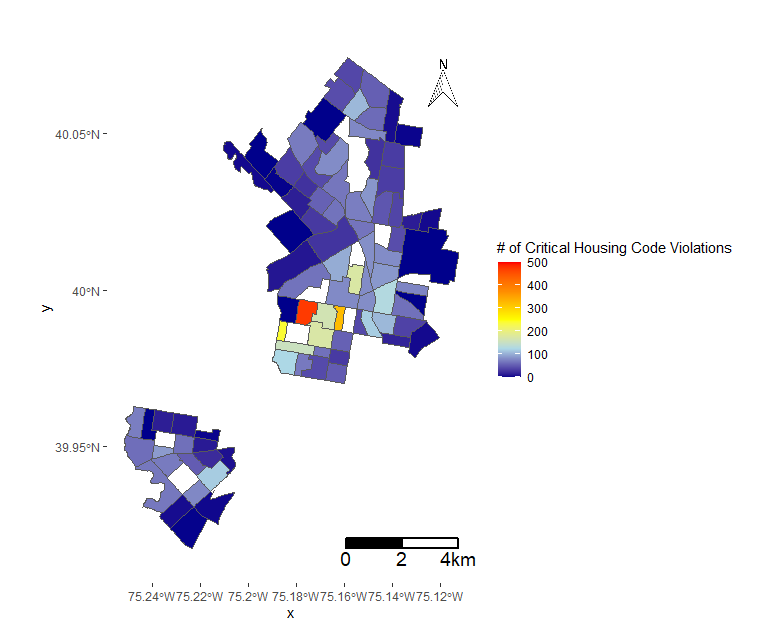
**

**Figure S12.** Critical housing code violations by census tract for the region of study.

**
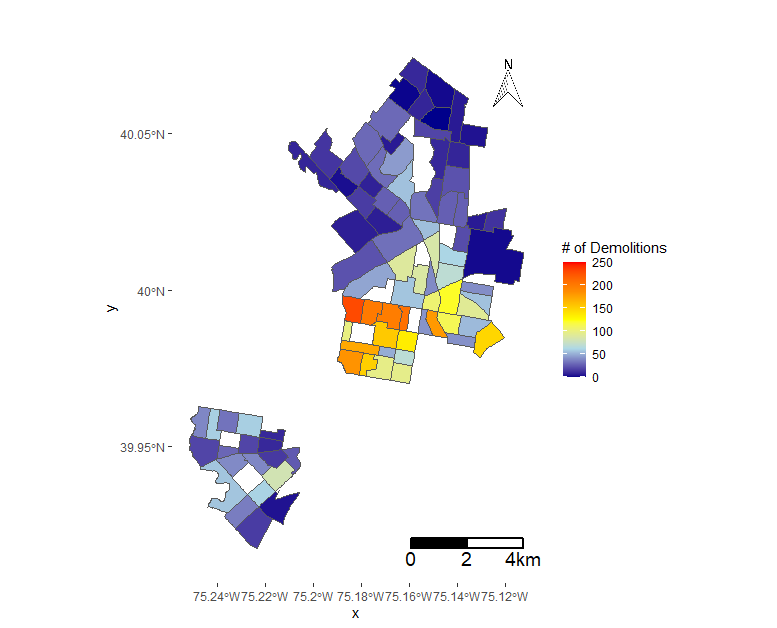
**

**Figure S13.** Demolitions by census tract for the region of study.

**
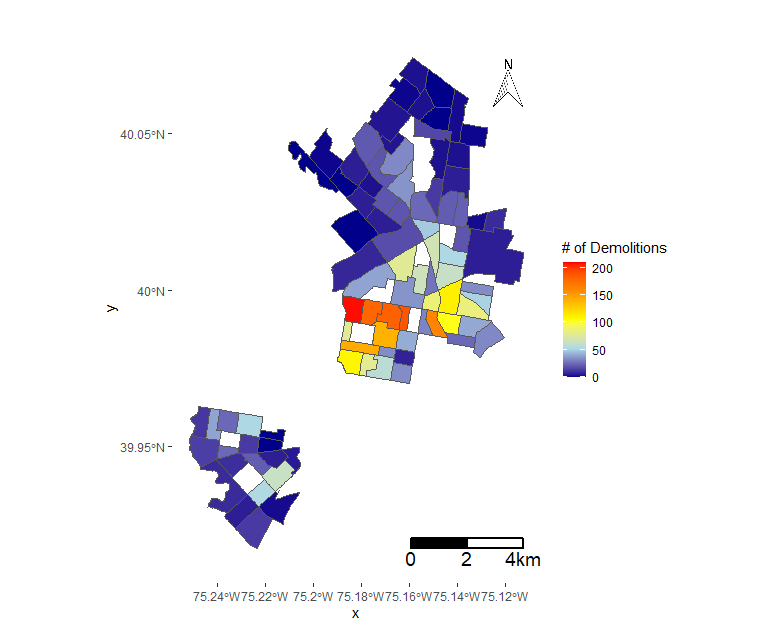
**

**Figure S14.** Demolitions due to housing code violations by census tract for the region of study.

**
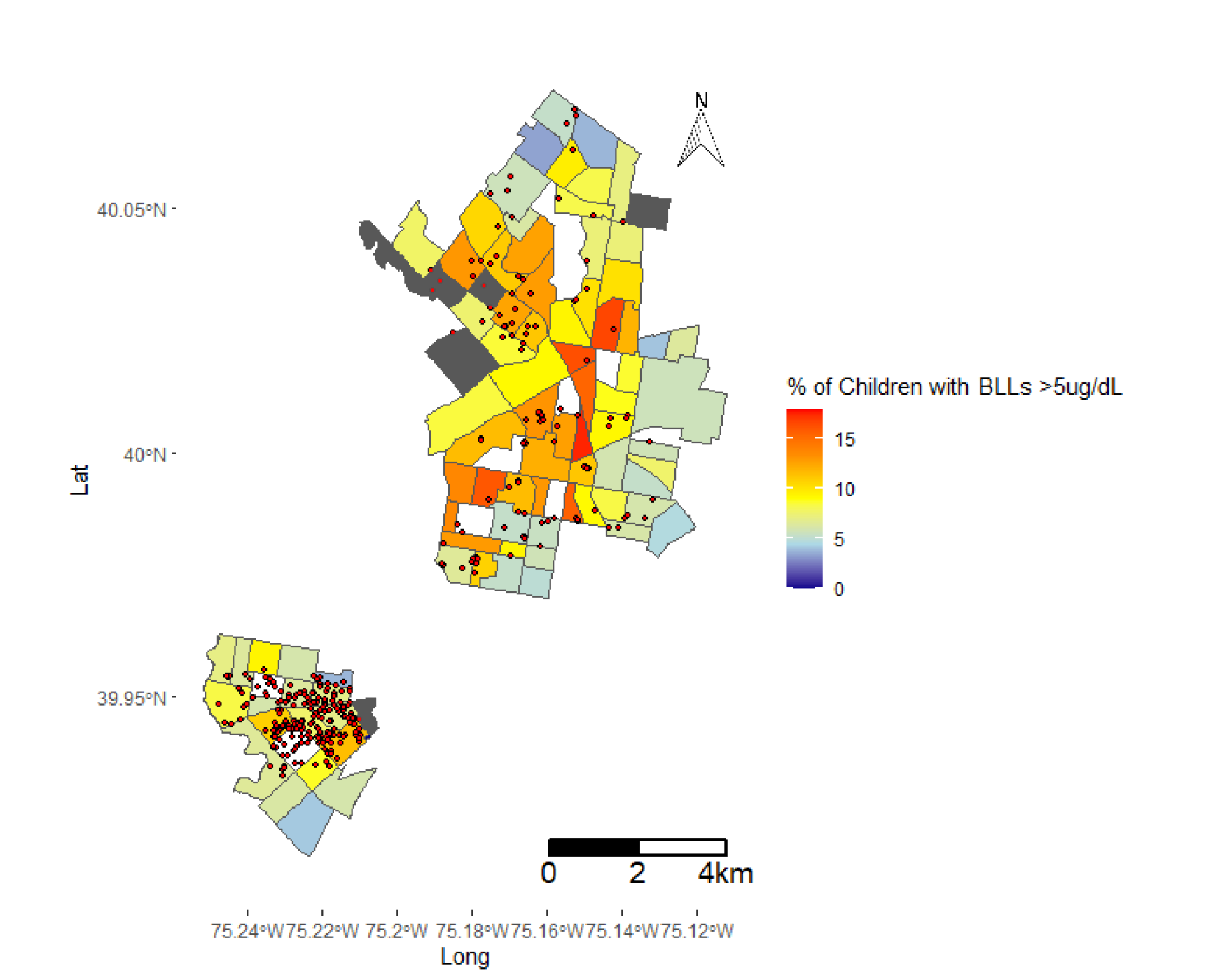
**

**Figure S15.** Lead-in-soil data points (dots) overlayed on the map showing the percentage of children with elevated blood lead levels by census tract for the region of study.

**
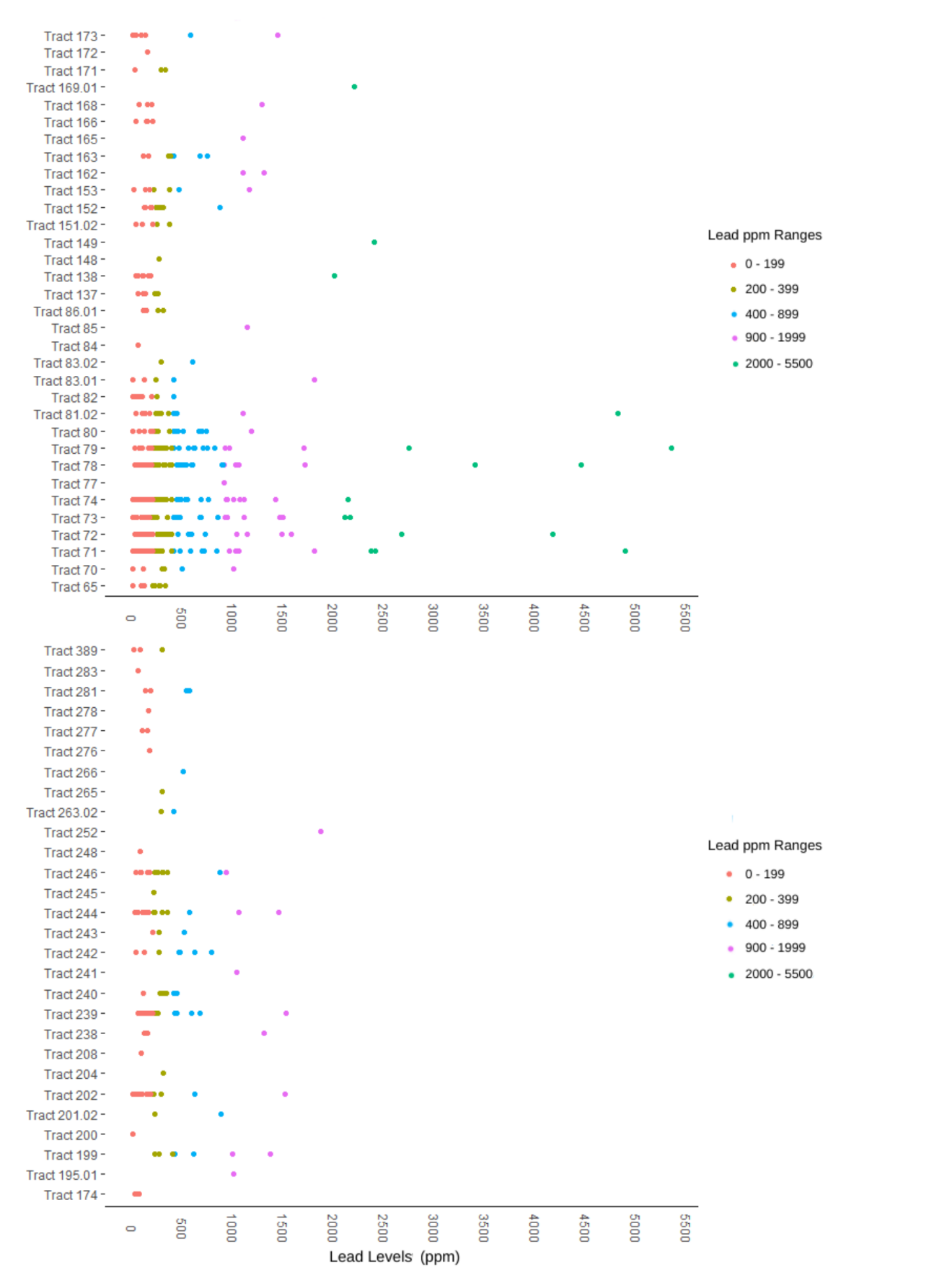
**

**Figure S16.** All 570 soil points collected in each of the studied census tracts with their respective concentration of lead in ppm.


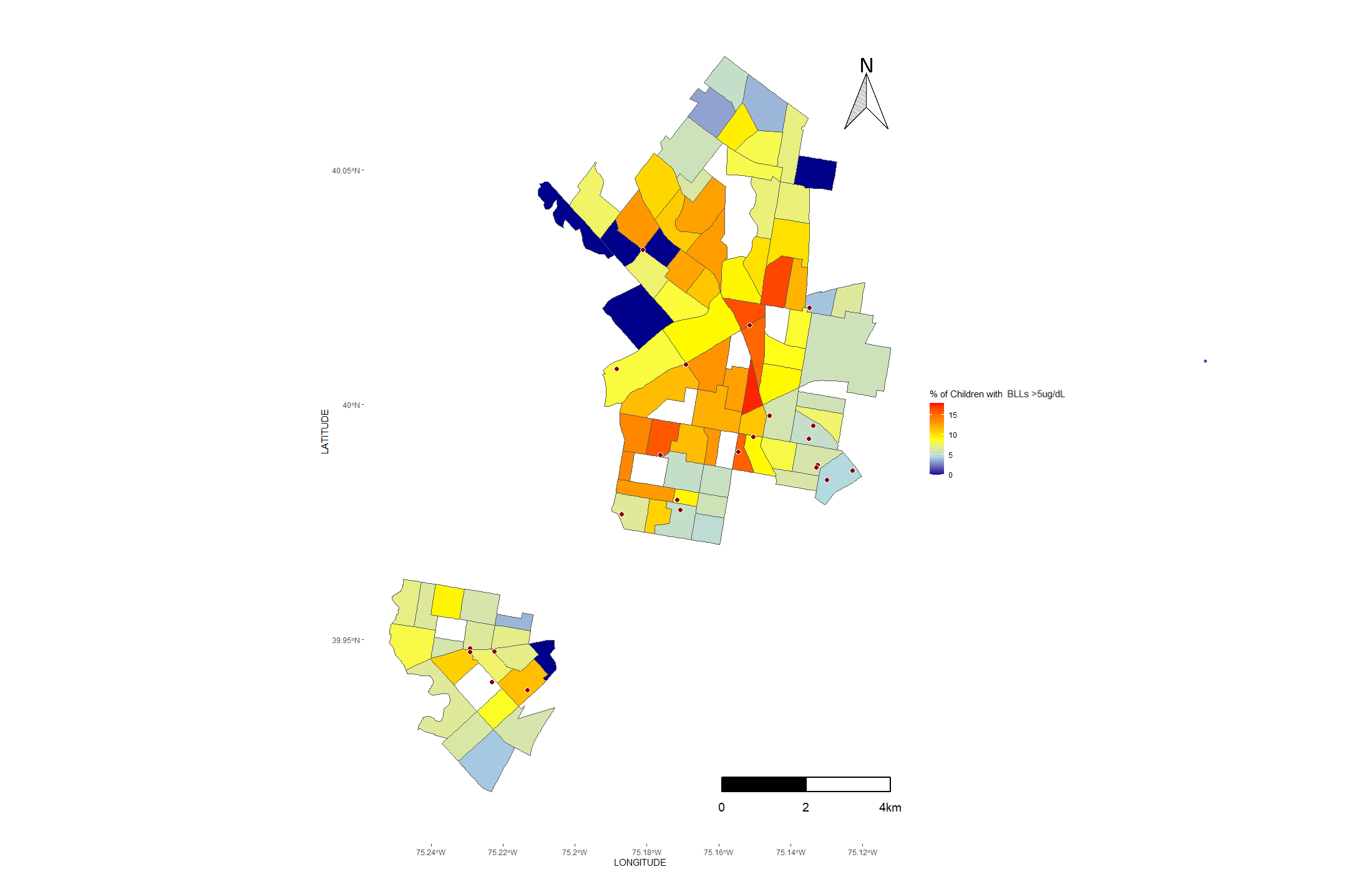


**Figure S17.** Land recycled sites (red dots) overlayed on elevated blood lead levels of children by census tract for the region of study.


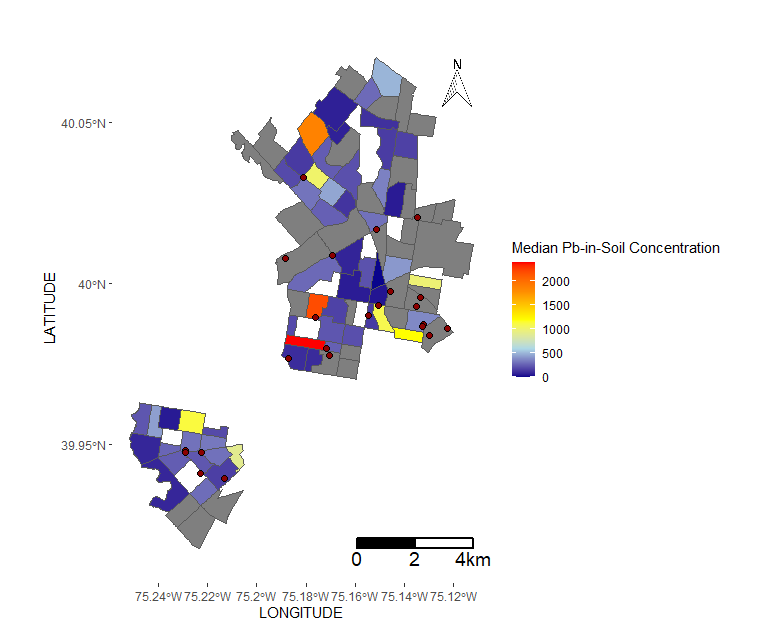


**Figure S18.** Land recycled sites (red dots) overlayed on median lead-in-soil data. Areas with high concentrations of hazardous soil lead levels are represented by bright red, whereas census tracts with no hazardous soil data are presented by dark blue.

**
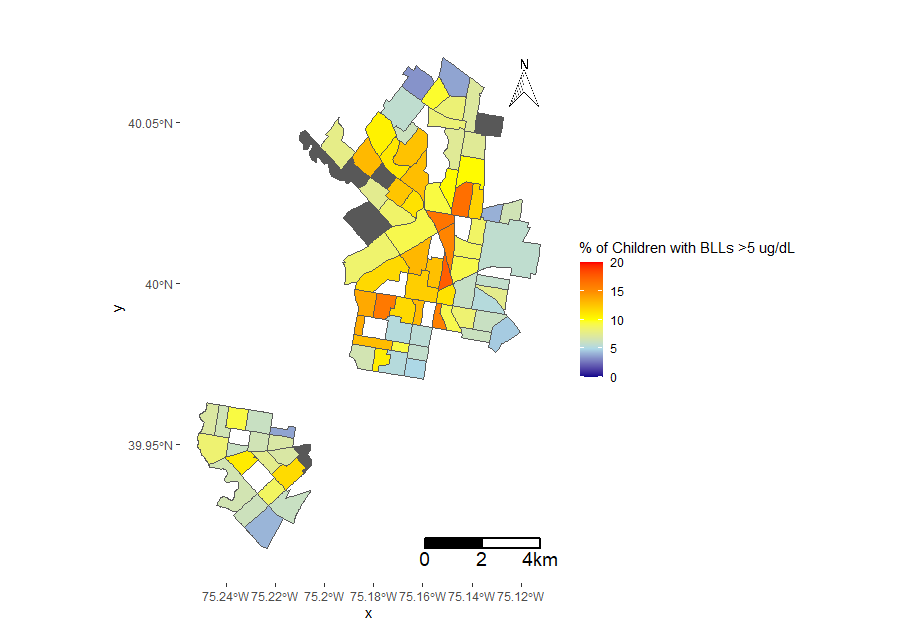
**

**Figure S19.** Elevated blood lead levels of children by census tract for the region of study.


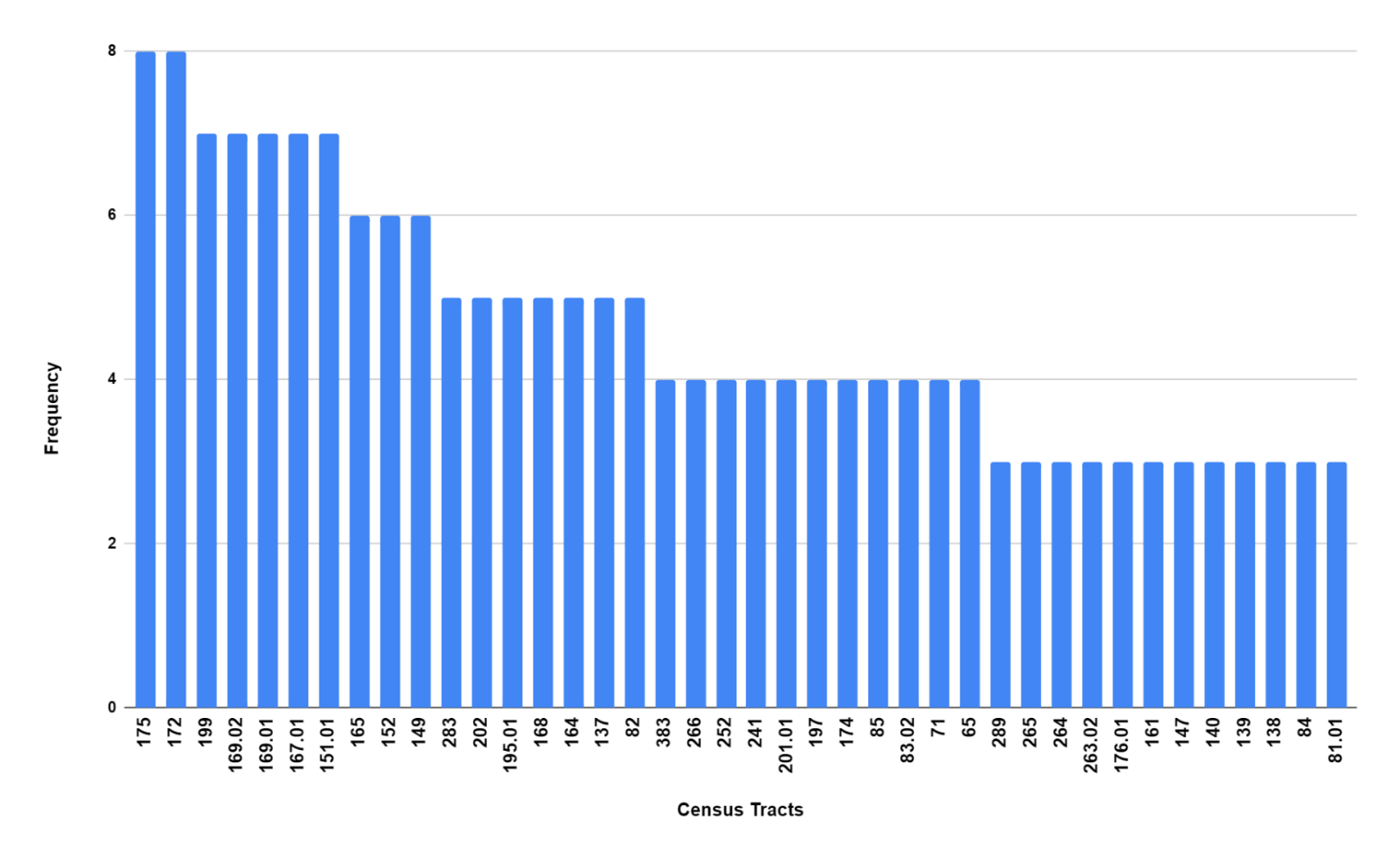


**Figure S20.** The number of times at which census tracts appeared as high risk among the thirteen lead-risk factors identified within this research. The thirteen lead-risk factors were as follows: 2007-2020 housing code violations, 2007 - 2020 critical housing code violations, 2007-2020 housing lead violations, 2015 data on children with EBLLs, 2007-2020 demolitions, 2007-2020 demolitions due to a violation, properties built before 1980, properties built before 1950, historical smelters, hazardous soil lead level data, children in poverty, median income, and minority population.


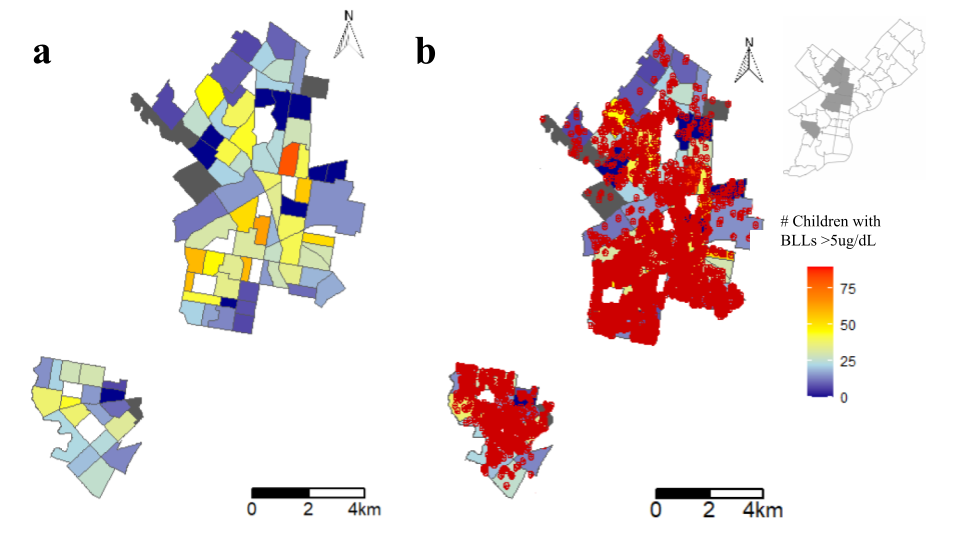


**Figure S21.** a) Map showing the absolute number of children with elevated blood lead levels by census tract; b) map showing the demolition sites and their radius of impact overlayed on the map a). At the top right corner is a visual of the region of study within Philadelphia.


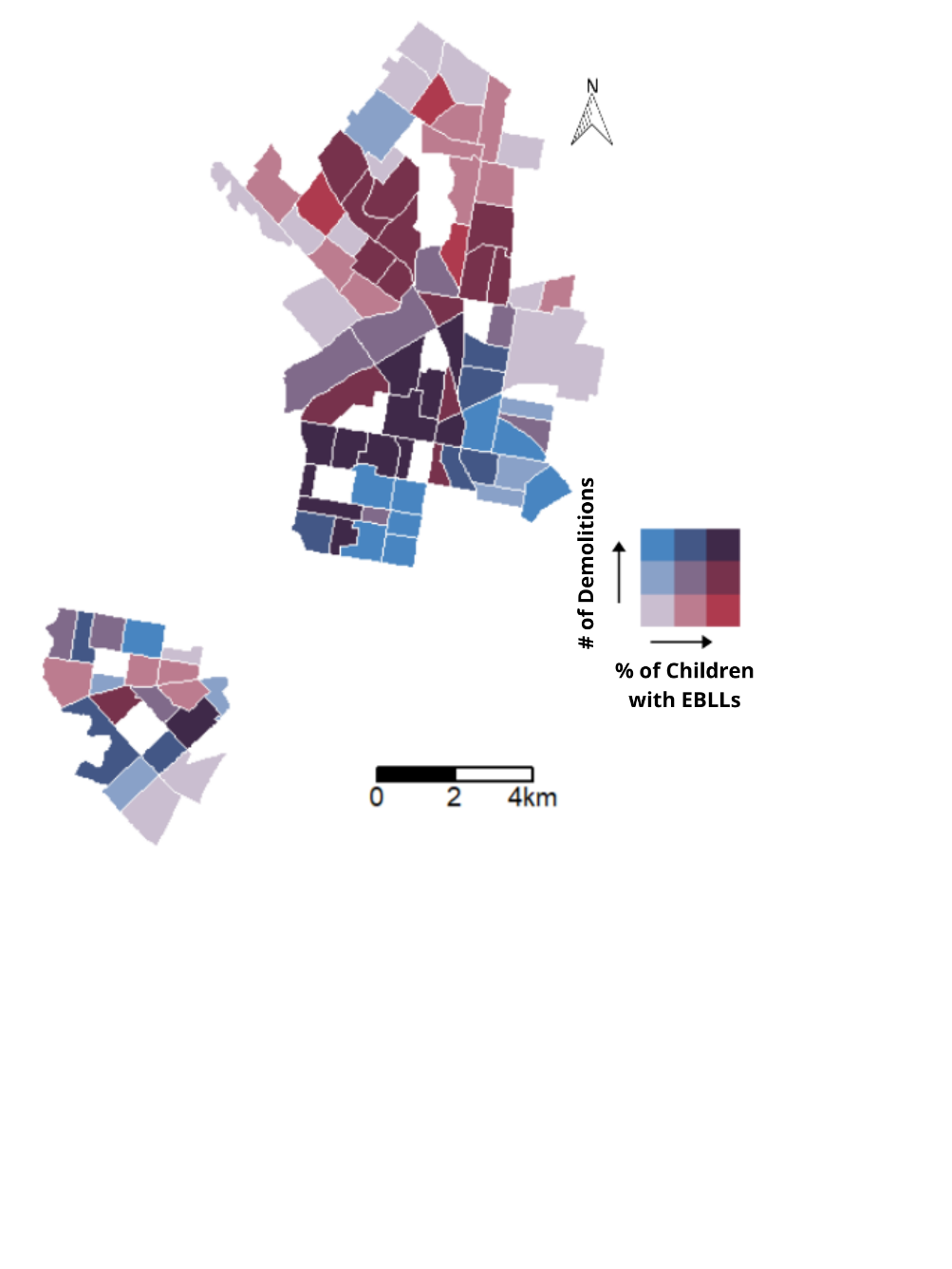


**Figure S22.** Bivariate map of the number of demolitions and percent of children with elevated blood lead levels by census tract for the region of study.


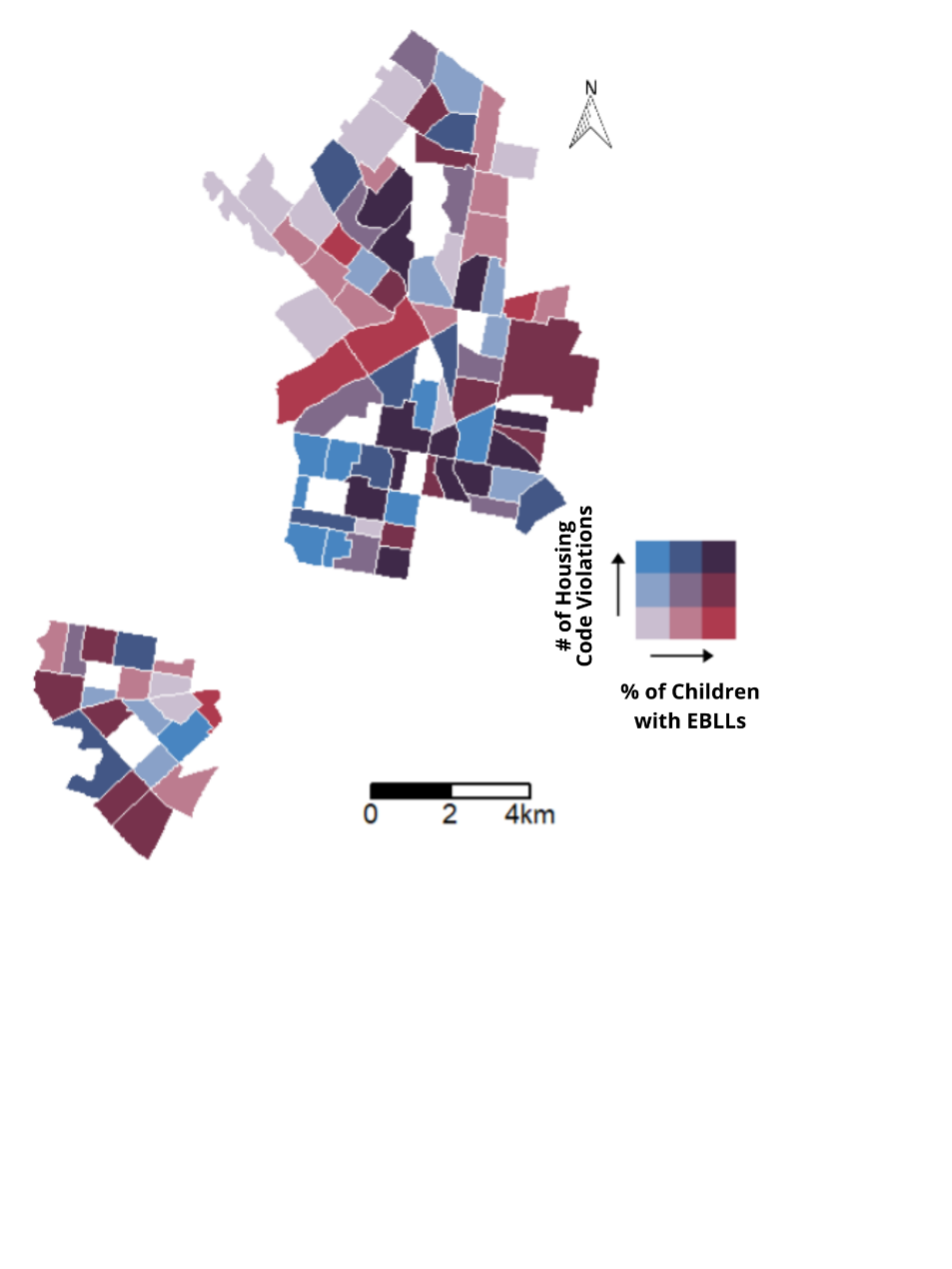


**Figure S23.** Bivariate map of percent of children with elevated blood lead and number of housing code violations by census tract for the region of study.


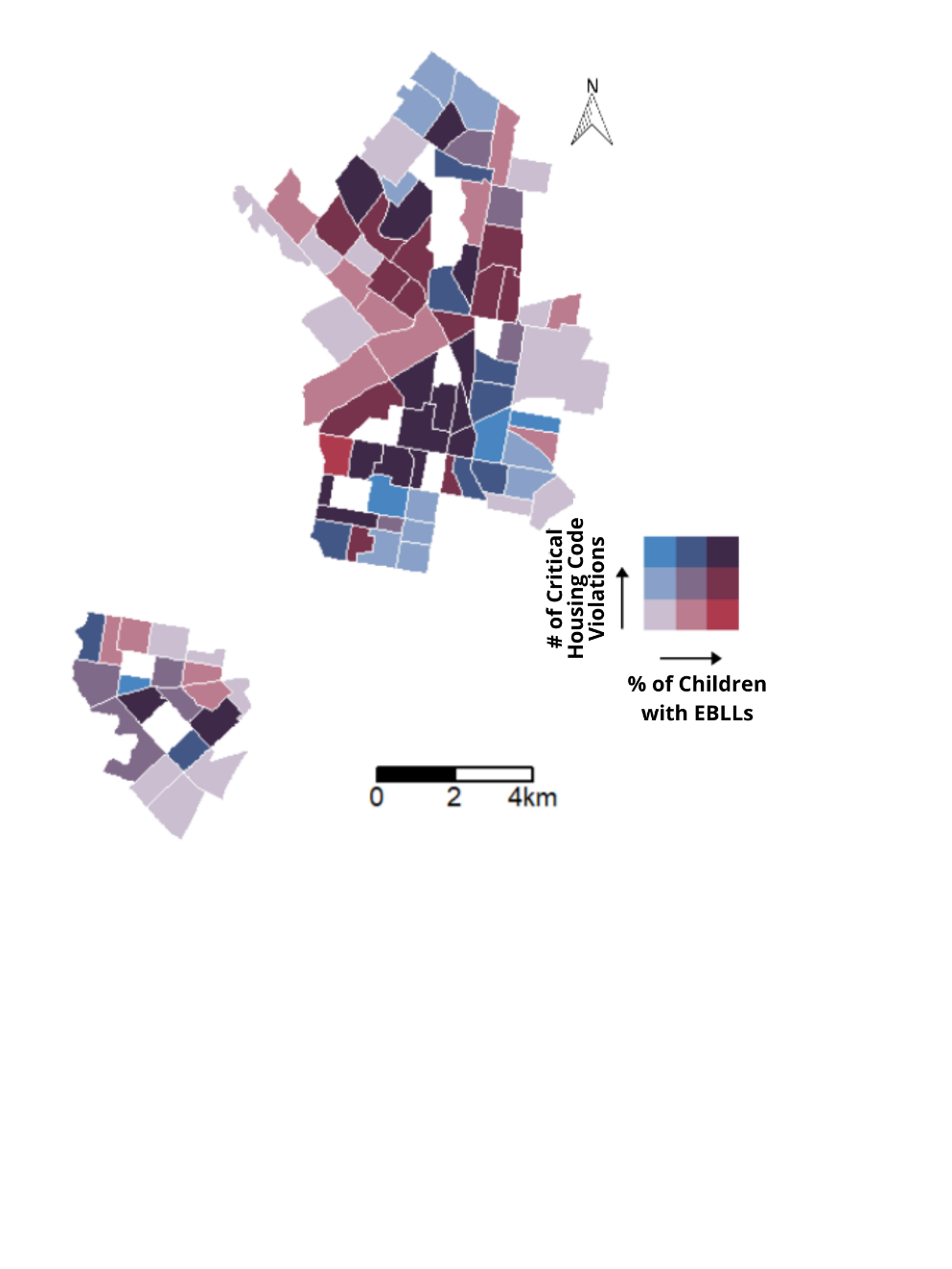


**Figure S24.** Bivariate map of the percent of children with elevated blood lead levels and number critical housing code violations by census tract for the region of study.


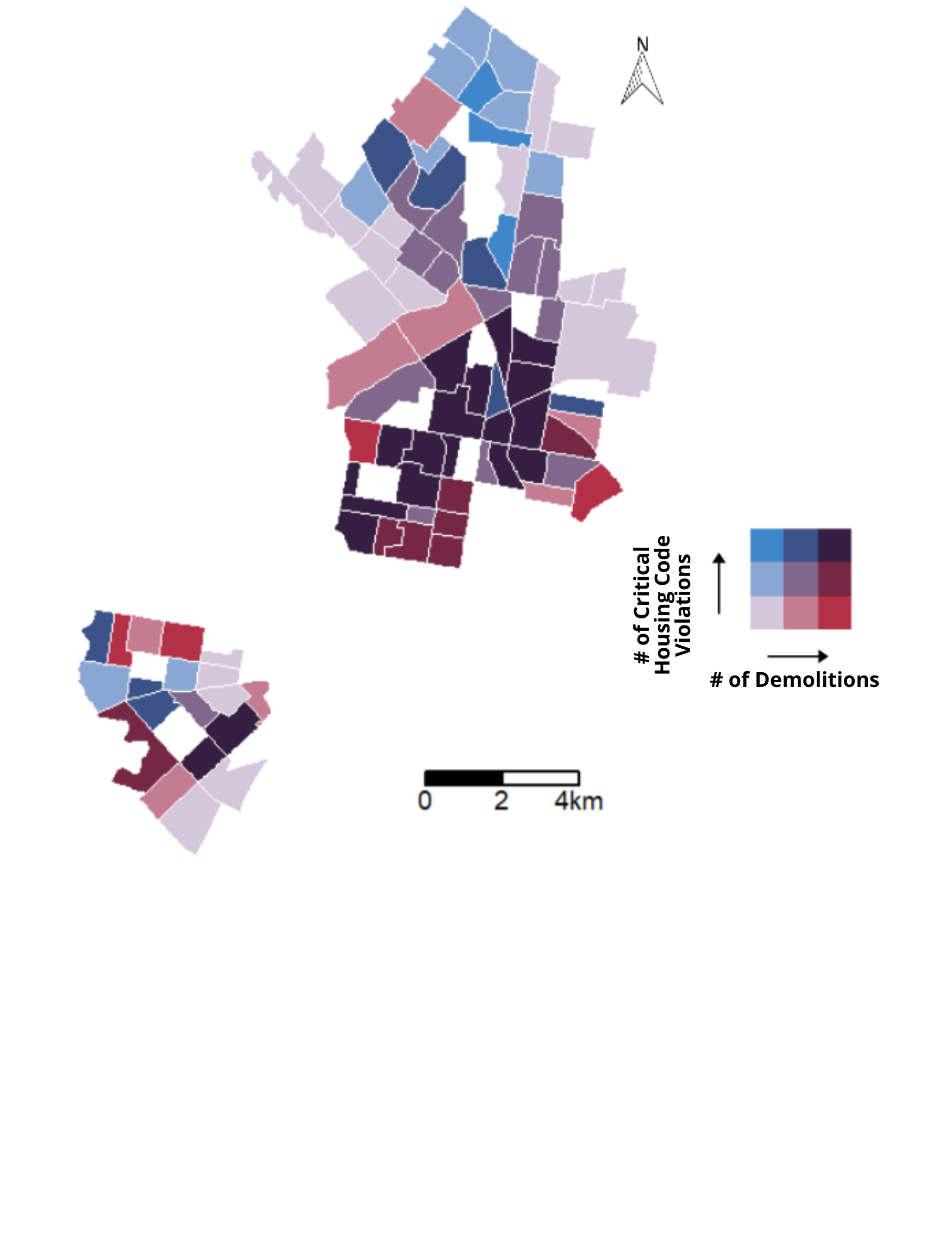


**Figure S25.** Bivariate map of the number of critical housing code violations and demolitions by census tract for the region of study.


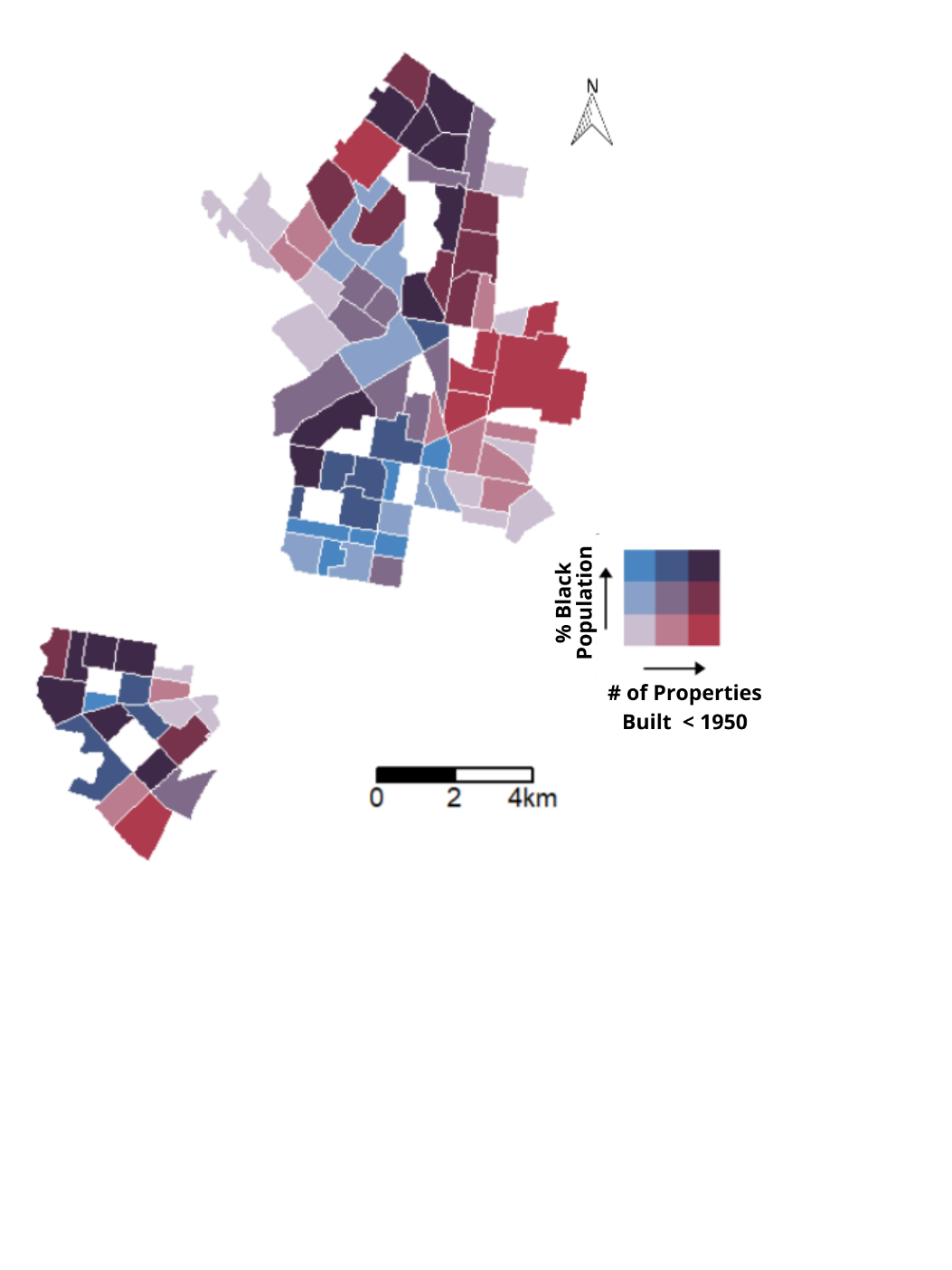


**Figure S26.** Bivariate map of the percent Black population and number of properties built before 1950 by census tract for the region of study.


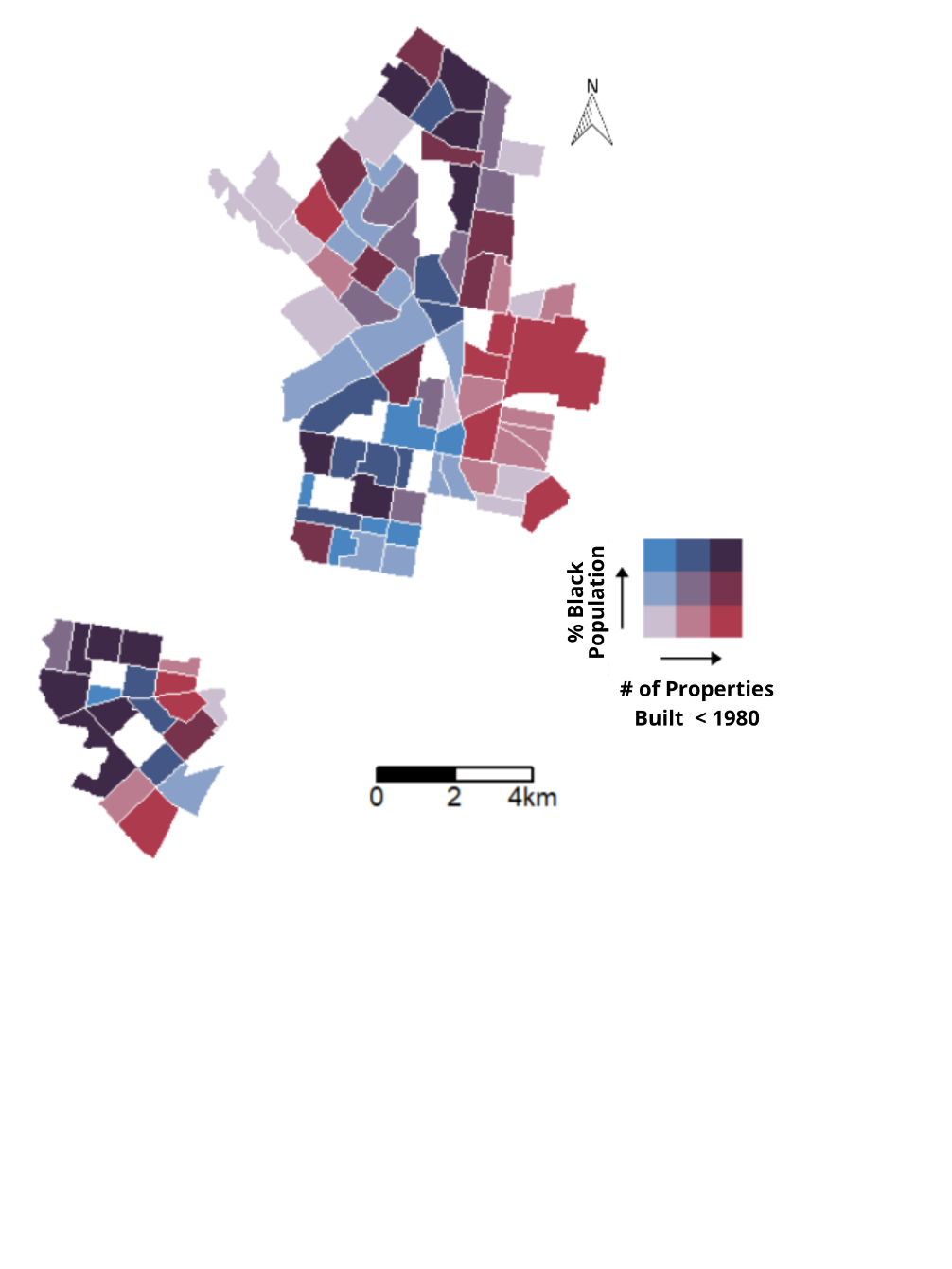


**Figure S27.** Bivariate map of the percent Black population and number properties built before 1980 by census tract for the region of study.


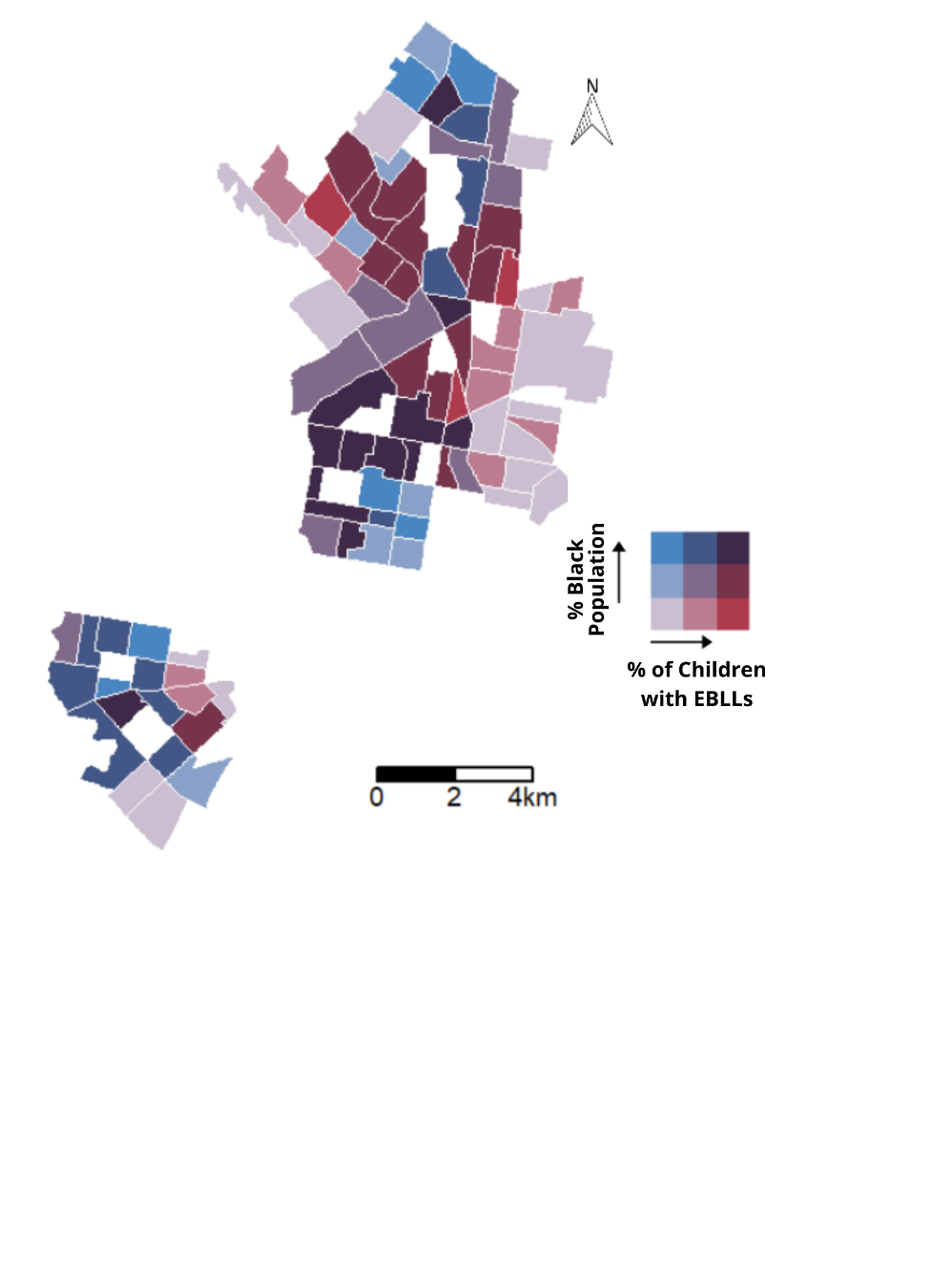


**Figure S28.** Bivariate map of the percent Black population and percent of children with elevated blood lead levels by census tract for the region of study.


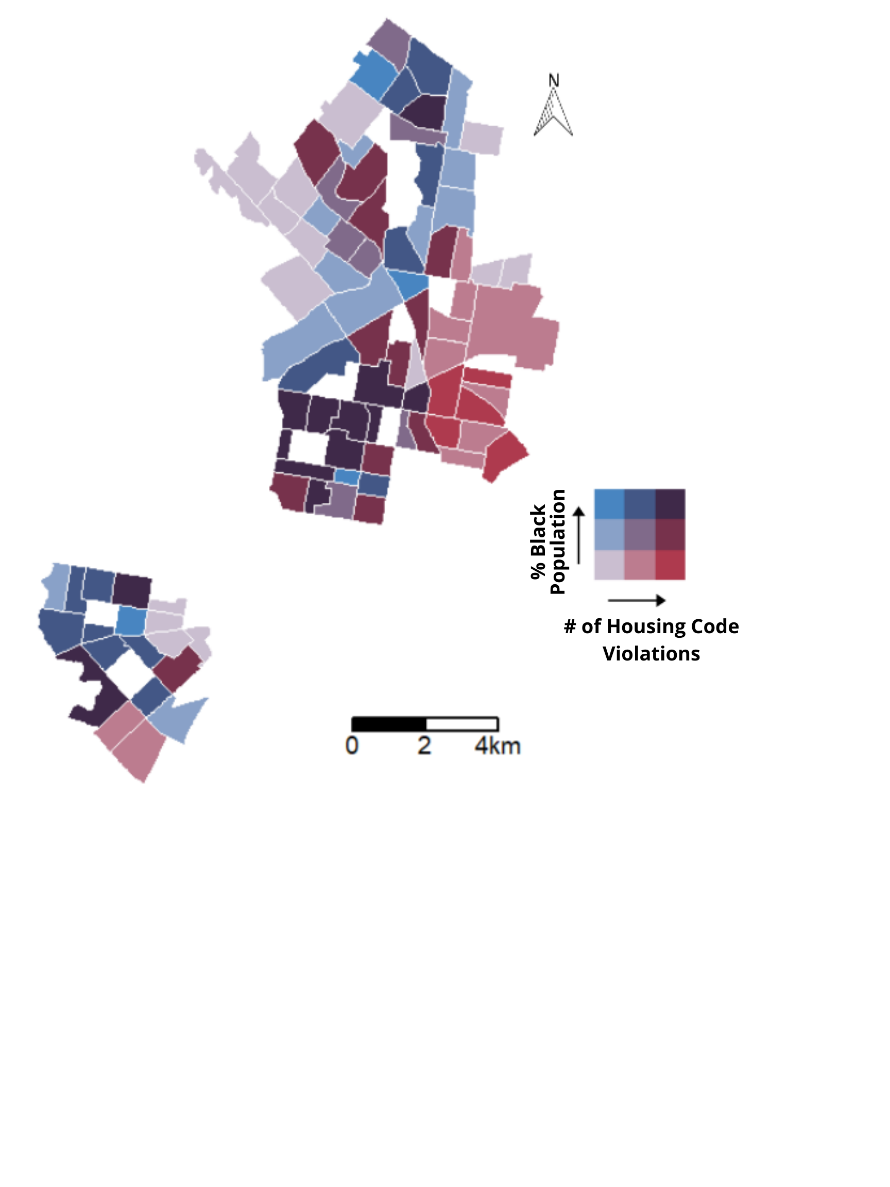


**Figure S29.** Bivariate map of the percent Black population and number of housing code violations by census tract for the region of study.

**
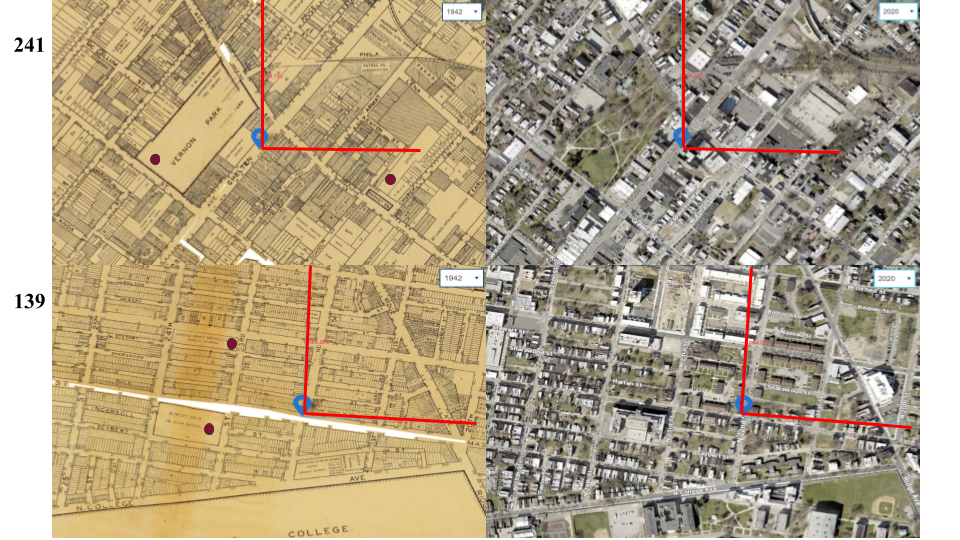
**

**Figure S30.** Historic smelter sites in census tracts 241 and 139 (blue markers) and each sites’ 500-meter radius of impact (red lines). The aerial views of the sites are provided both in 1942 and 2020 to visualize the open land where lead may have settled and still be in today. Red dots indicate open land areas for future sampling.

**
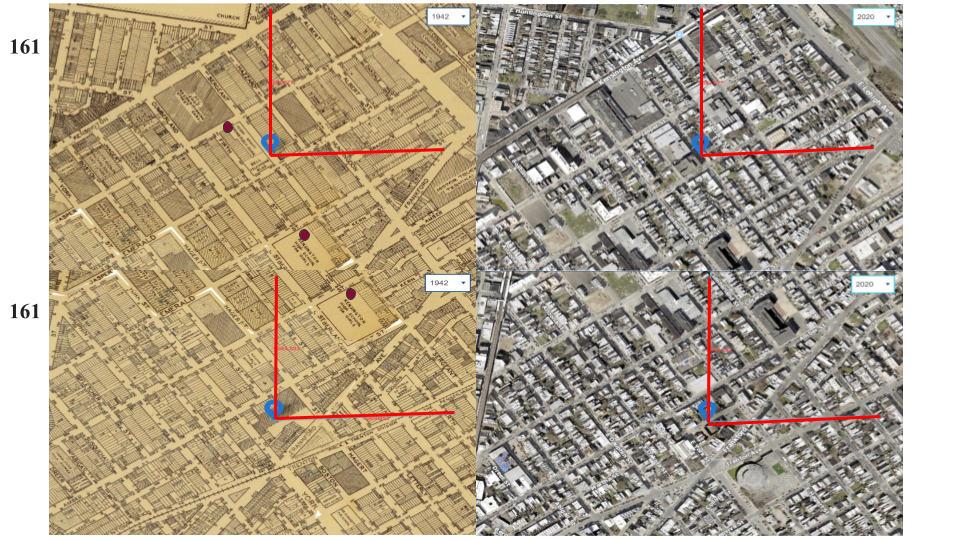
Figure S31.** Historic smelter sites in census tract 161 (blue markers) and each sites’ 500-meter radius of impact (red lines). The aerial views of the sites are provided both in 1942 and 2020 to visualize the open land where lead may have settled and still be in today. Red dots indicate open land areas for future sampling.

Table S1. Identified high lead-risk zip codes alongside census tracts found within and along the border of each.

| High Lead-Risk Zip codes | Census Tracts |
| --- | --- |
| 19121 | 140, 139, 138, 137, 149, 148, 147, 153, 152, 151.01 |
| 19132 | 166, 167.01, 167.02, 168, 169.01, 169.02, 170, 171, 172.01, 172.02, 173, 174, 200, 201.01, 202, 9800 |
| 19140 | 171, 173, 175, 195.01, 195.02, 197, 198, 199, 201.01, 202, 203, 204, 205, 280, 281, 283, 284, 287, 288, 289, 383, 9805 |
| 19144 | 206, 235, 236, 238, 239, 240, 241, 242, 243, 244, 245, 246, 247, 248, 252, 279.02, 280, 389, 9801 |
| 19143 | 65, 66, 67, 69, 70, 71.01, 71.02, 72, 73, 74, 77, 78, 79, 80, 81.01, 82, 83.01, 83.02, 84, 85, 86.01, 9809 |
| 19141 | 268, 270, 276, 277, 278, 279.01, 280, 281, 282, 283, 284 |
| 19133 | 161, 162, 163, 164, 165, 175, 176.01, 176.02 |
| 19138 | 252, 263.02, 264, 265, 266, 267, 277, 389 |

Table S2. Spearman correlation coefficient values between the lead-risk variables evaluated in this research. The Spearman correlation coefficients were calculated between all lead-risk factors, but only the significant ones (p < 0.05) are listed here. All other coefficients are available in the data repository.

| **Lead-Risk Variables** | **Spearman Correlation Coefficient R** |
| --- | --- |
| Owner Occupied Units to Properties Built Before 1980 | 0.78 |
| Demolitions to Housing Code Violations | 0.74 |
| Demolitions due to Violations to Housing Code Violations | 0.74 |
| Children with EBLLs to Housing Code Violations | 0.68 |
| Properties Built Before 1980 to Minority Population | 0.69 |
| Properties Built Before 1980 to Black Population | 0.66 |
| Median Income to Demolitions | -0.65 |
| Median Income to Demolitions due to Housing Code Violations | -0.65 |
| Children with EBLLs to Demolitions due to Housing Code Violations | 0.64 |
| Demolitions due to Violation to Critical Housing Code Violations | 0.63 |
| Demolitions to Critical Housing Code Violations | 0.58 |
| Children with EBLLs to Critical Housing Code | 0.58 |
| Properties Built Before 1950 to Minority Population | 0.57 |
| Owner Occupied Units to Black Population | 0.56 |
| Children with EBLLs to Demolitions | 0.55 |
| Properties Built Before 1950 to Black Population | 0.54 |
| Children with EBLLs to Black Population | 0.53 |
| Children with EBLL to Minority Population | 0.51 |
| Black population to Housing Code Violations | 0.50 |

**Data Set S1.** The dataset provides all data used for the creation of the maps and spearman correlation analysis, along with the full spearman correlation analysis done. This is all available at The Environmental Data Initiative via the DOI: https://doi.org/10.6073/pasta/6844ce2d54f15c2b5a1ed4d57b819eda which is released to the public domain under Creative Commons CCO 1.0.
